# CRISPR-Cas Targeting of Host Genes as an Antiviral Strategy

**DOI:** 10.3390/v10010040

**Published:** 2018-01-16

**Authors:** Shuliang Chen, Xiao Yu, Deyin Guo

**Affiliations:** 1 School of Basic Medical Sciences, Wuhan University, Wuhan 430071, China; Chen-shuliang@whu.edu.cn; 2 Institute of Health Inspection and Testing, Hubei Provincial Center for Disease Control and Prevention, Wuhan 430079, China; fish-yuxiao@hotmail.com; 3 School of Medicine (Shenzhen), Sun Yat-sen University, Guangzhou 510080, China

**Keywords:** gene targeting, CRISPR-Cas, host genes, virus, antiviral strategy

## Abstract

Currently, a new gene editing tool—the Clustered Regularly Interspaced Short Palindromic Repeats (CRISPR) associated (Cas) system—is becoming a promising approach for genetic manipulation at the genomic level. This simple method, originating from the adaptive immune defense system in prokaryotes, has been developed and applied to antiviral research in humans. Based on the characteristics of virus-host interactions and the basic rules of nucleic acid cleavage or gene activation of the CRISPR-Cas system, it can be used to target both the virus genome and host factors to clear viral reservoirs and prohibit virus infection or replication. Here, we summarize recent progress of the CRISPR-Cas technology in editing host genes as an antiviral strategy.

## 1. Introduction

### 1.1. The Development of CRISPR-Cas System

#### 1.1.1. Canonical CRISPR-SpCas9

The Clustered Regularly Interspaced Short Palindromic Repeats (CRISPR)-associated (Cas) system was first discovered as an immune response against bacteriophage infections and invading plasmids in prokaryotes such as Bacteria and Archaea [1,2]. The CRISPR-Cas immune system involves the helicase—called the Cas protein—which can bind to RNA transcribed from palindromic repeats of DNA or cleaved DNA paired with RNA spacers—which are transcripts from the short stretches of DNA acquired from extra chromosomal elements [3,4]. Specifically, in the most used type II CRISPR-Cas system, the transcript of palindromic repeats of DNA is called the transactivating CRIPSR RNA (tracr RNA), while the spacers’ transcript is named CRIPSR RNA (crRNA) [5]. The tracr RNA and crRNA can be linked together to form a single guide RNA (sgRNA) that can direct Cas9 to induce a double stranded DNA break in the Protospacer adjacent motifs (PAMs) region [6,7]. In doing so, the cleavage stimulates non-homologous end joining (NHEJ) or homology-directed repair (HDR)-mediated genome editing, thus causing insertion or deletions (indels) in the genome of cells [8]. After initial confirmation of CRISPR-SpCas9 system-mediated genome editing in eukaryote mammalian cells, a series of Cas9 mutants or analogues and multi CIRSPR-Cas delivery vectors have been developed—such as lentiviral (LV), adenoviral (AV), as well as adeno associated viral (AAV) plasmids—ultimately to broaden its application in various research fields [9,10,11].

#### 1.1.2. CRISPR Interference (CRISPRi)

Crystal structure studies of Cas9 bound to sgRNA and target DNA revealed that Cas9 protein harbors two important nuclease domains, RuvC and histidine-asparagine-histidine (HNH), which are essential for cleavage of the complementary and non-complementary strands of the target DNA [5,12,13]. Mutations in the RuvC (D10A) and HNH (H840A) domain of SpCas9 allow it to be converted into a DNA “nickase” that cleaves a single strand break and induces specific non-homologous end joining (NHEJ) and homologous recombination (HR) [5]. Inactive Cas9 (dCas9, spCas9D10A/H840A) can bind to sgRNA and target DNA without cleavage, which is a versatile tool applied in regulation of gene expression. Qi et al. named it CRISPR interference (CRISPRi) technology, which can efficiently inhibit expression of target genes in *Escherichia coli* as well as in mammalian cells by the transcription block of the elongating ribonucleic acid polymerase (RNAP) with dCas9:sgRNA complex [14]. However, this CRISPRi can only achieve 2 fold decrease of gene expression in human cells. To improve the efficacy of CRISPRi, they fused dCas9 with a repressive chromatin modifier domain, Krüppel associated box (KRAB) from Kox1 protein. This artificial dCas9-KRAB further recruits chromatin-modifying complexes to silence transcription of the sgRNA targeted genes [15].

#### 1.1.3. CRISPR Activation (CRISPRa)

In comparison to CRISPRi, which inhibits gene expression, the combination of four copies of the transcription activator VP16 (Virus Protein 16 is a transcription factor encoded by the UL48 gene of Herpes simplex virus-1) or a single copy of p65 activation domain (AD) with dCas9 (dCas9-VP64 and dCas9-p65AD) can trans-activate gene transcription in cells [15]. In addition, single or multiple gRNAs targeting the gene promoter to direct dCas9-VP64 activation of the transcription of endogenous human genes was exploited. It was found that gRNAs targeting multiple loci in the same promoter have synergistic effects on activation of gene expression [16,17]. Thereafter, Zhang’s group described a structure guided optimization of the CRISPR-Cas9 complex to induce efficient transcriptional activation of up to ten endogenous genes at one time. This system contains a previously reported dCas9-VP64 fusion protein and an engineered sgRNA containing substitutions in the tetraloop and stem loop 2 domains of two minimal hairpin aptamers that selectively interacts with dimerized MS2 bacteriophage (a bacterial virus) coat protein, the MS2 fused with the NF-κB trans-activating subunit p65 and the activation domain from human heat-shock factor 1 (HSF1). The transcription activator VP64 can recruit a distinct subset of transcription factors such as PC4, CBP/p300 and the SWI/SNF complex, which is originally defined in yeast as the protein complex important for cellular responses to mating-type switching (SWI) or sucrose fermentation (SNF)). p65 can recruit AP1, ATF/CREB and SP1, whereas HSF1 further improves the transcriptional complex activity. The triple combination of sgRNA2.0, MS2-p65-HSF1 and dCas9-VP64 comprises the most effective synergistic activation mediator (SAM) system. In addition, the sgRNA that targets to 200 bp upstream of the transcription start site has the most potential activation efficacy [18].

In addition to the SAM system, Tanenbaum et al. developed another dCas9-SunTag-VP64 system that significantly improved the expression of endogenous genes. In the dCas9-SunTag-VP64 CRISPRa system, synthetic transcriptional activator VP64 is fused to green fluorescent protein (GFP) and single chain variable fragment (scFv) antibodies with binding specificity for peptides derived from the general control protein 4 (GCN4). The dCas9 fused to GCN4-containing peptide tags (Sun-Tag) can recruit up to 24 copies of GFP, leading to spatial recruitment of multiple VP64 domains to the gRNA-complementary target sites. Therefore, the dCas9-24xGCN4-v4, scFv-GCN4-sfGFP-VP64 and sgRNA constitutes the dCas9-SunTag-VP64 system provides a versatile toolset for gene activation [19].

#### 1.1.4. Non Canonical CRISPR-Cas System-SaCas9, Cpf1, FnCas9, C2c1/2/3 and CRISPR-Cas13

##### SaCas9

Since the full-length of SpCas9 is about 4.5 kb, the large size limits its usage in basic research and therapeutic applications to adenoviral or lentiviral vectors as a delivery tool. However, the lentiviral vector can be integrated into host genome and has a potential risk factor for induction of carcinogenesis, whereas the adenoviral vector has strong immunogenic effect in human cells. In order to identify a more versatile genome-editing platform with smaller cargo size, Ran et al. found a *Staphylococcus aureus* (SaCas9) that is 1 kb shorter compared to SpCas9. This SaCas9 can be packaged in to the highly versatile adeno-associated virus (AAV) delivery vector, which is broadly used for clinical research due to the broad range of serotype specificity, reduced oncogenic risk from host-genome integration and low immunogenic potential [20]. However, there is a drawback concerning re-administration of AAV vectors because of immunogenicity and consequently reduced re-delivery of AAV vectors [21,22]. Aside from the NNGRRT PAMs targeting site for wild type SaCas9, Kleinstiver et al. generated a SaCas9 (KKH) variant that harbors more broad target recognition sites with NNA(C/T)RRT PAMs [23]. Because of the tremendous promise for biomedical research, SaCas9-mediated genome editing has been successfully applied in plant, zebra fish and mouse models [24,25,26]. Furthermore, the crystal structures of SaCas9 in complex with sgRNA and target DNA were reported, which provides a structural information based design of CRISPRi or CRISPRa that uses dSaCas9 and expands the genome editing choice [27].

##### Cpf1

Cpf1 is assigned to the class 2 type V CRISPR system. Zhang’s group first experimentally confirmed its application as a genome editing tool in mammalian cells. Distinct from both SpCas9 and SaCas9, Cpf1 is a single RNA-guided endonuclease without tracrRNA and recognizes PAMs of 5′-TTN-3′ or 5′-CTA-3′downstream of the protospacer. These characteristics enable Cpf1 cleavage of A/T-rich genomes with only a single crRNA of 42–44 nucleotides long (23–25-nucleotide spacer and 19-nucleotide repeat). By testing 16 Cpf1-family proteins from various bacteria, only two Cpf1 enzymes from *Acidaminococcus sp. BV3L6* (AsCpf1) and *Lachnospiraceae bacterium ND2006* (LbCpf1) were found to have the ability to manipulate the genome in human cells [28,29]. After solving the crystal structure of AsCpf1, a five amino acid residues mutant (H800A, K809A, K860A, F864A and R790A) was developed to target up to 4 genes in mammalian cells and 3 in the mouse brain, using an AAV cassette containing a single target array of multiplex crRNA and the nuclear localization signal (NLS) fused AsCpf1 [30,31]. The simplicity of crRNA design and high efficiency of genome editing enable Cpf1 to exert a genetic operation in a variety of organisms, including cyanobacteria, rice, tobacco and mice [32,33,34,35]. In addition to DNA cleavage, Cpf1 from *Francisella novicida* (FnCpf1) also has RNase activity to process pre-crRNA to mature crRNAs in vitro and can be designed for RNA genome targeting [36]. The dual DNase and RNase activities of Cpf1 should be considered when designing lentiviral vectors for expression of crRNA and nucleases [31]. However, the Cpf1 targeting RNA in mammalian cells was not reported and need further research.

##### FnCas9

Cas9 of *F. novicida* (FnCas9) is encoded by the *FTN_0757* gene and was predicted to be a component of the CRISPR/Cas system by bioinformatic analysis. Distinct from typical CRISPR/Cas systems that induce foreign DNA breakage and degradation, FnCas9 modulates endogenous anti-bacterial lipoprotein FTN_1103 (BLP) expression by degrading its mRNA, thus facilitating bacterial evasion of this innate immune defense system and increasing virulence [37]. Because FnCas9, tracrRNA and scaRNA contribute to virulence, bacterial mutants of these components can be designed as an attractive vaccine strain for other pathogens. Based on these findings, FnCas9 was used to target a human positive-sense single-stranded RNA (+ssRNA) virus, hepatitis C virus (HCV), through RNA guided HCV RNA genome degradation [38]. This research provides direct evidence that the versatile RNA-editing system FnCas9 can effectively function as an antiviral defense toolset in eukaryotic cells.

##### Class 2 Candidate 1/2/3 (C2c1/2/3)

CRISPR-Cas systems are generated by three steps; spacer formation, crRNA generation and genetic interference. According to the architecture of the interference modules, the CRISPR-Cas system can be divided into 2 groups: Class 1, containing types I, III and IV that utilize crRNA binding to multi-subunit effector complexes comprised of various Cas proteins; Class 2, including types II, V and VI, uses crRNA association with a single unit of Cas protein, such as the most studied Cas9 and CPF1. C2c1/2/3 (Class 2 candidate 1/2/3), according to the effector module with a single unit of crRNA, belongs to Class 2 type V CRISPR-Cas system [39]. The C2c1 and C2c3 harbor a RuvC-like endonuclease domain that breaks DNA, guided by tracrRNA, while the C2c2 contains two predicted higher eukaryote and prokaryote nucleotide-binding (HEPN) RNase domains which targets RNA [39]. Furthermore, C2c2 from the bacteria *Leptotrichia buccalis* (LbuC2c2), *Listeria seeligeri* (LseC2c2) and *Leptotrichia shahii* (LshC2c2) were experimentally confirmed as RNA-guided RNA-targeting CRISPR effectors that induce single stranded RNA (ssRNA) cleavage in vitro and in prokaryotes [40,41]. In addition, the detailed molecular principles of C2c1 and C2c2 CRISPR targeting were demonstrated by two crystal structure research groups using *Alicyclobacillus acidoterrestris C2c1* (AacC2c1) and LshC2c2 [42,43,44]. However, the mechanism of engineering them to edit eukaryotic cells is a challenge and should be explored in the future.

##### CRISPR-Cas13

Cas13 belongs to the class 2 type VI RNA-guided RNA nuclease and three proteins have been identified; Cas13a (C2c2), Cas13b and Cas13c. Cas13a from *Leptotrichia wadei* (LwaCas13a) can be engineered for targeting endogenous transcripts in human HEK293FT cells and in plants. Moreover, the monomeric superfolder GFP (msfGFP) fused LwaCas13a has more stability and specificity and can be developed for programmable tracking of transcripts in live cells [45]. A Cas13b ortholog from *Prevotella sp. P5-125* (PspCas13b), which is C-terminally fused to the nuclear export signal (NES) domain of HIV Rev gene, has the most efficient RNA interference in mammalian cells. Through mutation of the conserved catalytic residues in the HEPN domains, dPspCas13b, lacking nuclease activity, can be fused to the deaminase domain of ADARs (ADAR_DD_), which naturally deaminates adenosines to insosines in dsRNA and is engineered for modifying RNA, such as RNA editing for programmable A to I (G) replacement (REPAIR). Furthermore, to enhance the specificity of REPAIRv1 by utilizing a rational mutagenesis strategy, mutant ADAR2DD (E488Q/T375G) fused with dCas13b showed a precise, efficient and highly specific RNA targeting which was termed REPAIRv2 [46]. It offers an alternative RNA editing toolset for modifying the residues of proteins, thus altering their function in regulation of disease, such as autoimmune disorders including psoriasis, immunoglobulin A deficiency, type I diabetes and, systemic lupus erythematosus. However, the temporary nature of the REPAIR system restricts its application in diseases caused by temporary changes in cell state and the high off-target efficacy needs more attention in designing the target guide RNA.

### 1.2. Virus-Host Interactions and Strategies Blocking Virus Infection

**Figure 1:**
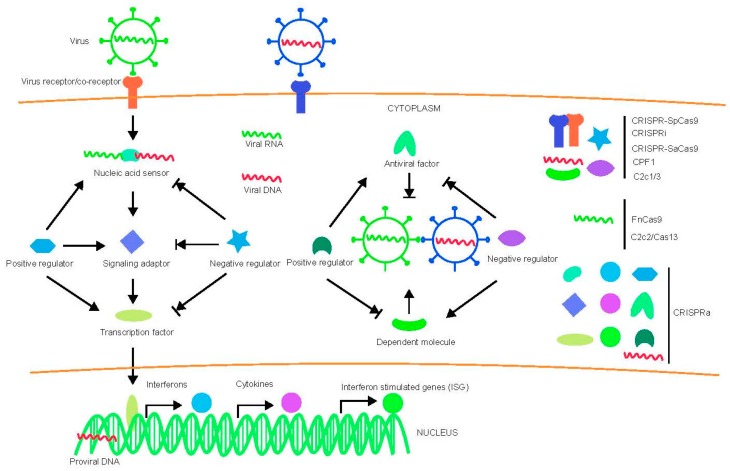
Diagram of virus-host interactions and possible targets for various CRISPR-Cas system. Arrow indicates promotion and arrow with short vertical line indicates inhibition.

Virus-host interactions are dynamic and persistent process. The virus exploits host factors to complete its life cycle and the host uses its immune defense system to inhibit or clear viral infection. In addition to the innate immune system and adaptive immune apparatus, host cells evolved to express general restriction factors that block virus replication. To counteract this, viruses use specific strategies to escape immune surveillance. Thus, CRISPR-cas systems to treat virus related diseases can be conducted by targeting viral nucleic acid, as well as host factors including positive and negative regulators as shown in Figure 1.

### 1.3. CRISPR-Cas System and Human Virus Restriction

Here, we compare different CRISPR-Cas systems and their application in anti-viral research by targeting host factors (Table 1). We also discuss recent progress in various CRISPR-Cas systems in targeting host factors to combat HIV, HBV, HCV, EBV, HSV, dengue virus, Ebola virus, SARS, MERS, Influenza virus and Zika virus. The potential host dependent and restriction factors targeted by CRISPR-Cas system are summarized in Table 2.

#### 1.3.1. Human Immunodeficiency Virus (HIV)

HIV-1 is a retrovirus and the viral genomic RNA will be converted into double-stranded DNA by HIV-1 reverse transcriptase [47,48]. After reverse transcription, the DNA inserts into the host cell genome to form provirus and can be activated to generate progeny viruses [49,50,51]. HIV-1 is the causative pathogen of acquired immunodeficiency syndrome (AIDS) which is a worldwide disease that can be effectively treated but a functional cure is not readily achievable [52,53]. It is a threat to humans due to the lack of an effective commercial vaccine. Individuals infected with HIV-1 rely on antiretroviral therapy (ART) treatment that limits the HIV-1 reservoir to a low level but has many drawbacks such as life-long treatment, high cost and chronic hepatic or cardiovascular system injury [54,55,56,57]. Gene therapy has become a possible method to cure AIDS since an American patient with leukemia lived free of HIV-1 infection after receiving a bone-marrow transplant from a tissue-matched donor with the homozygous *C-C chemokine receptor type 5 delta 32* (*CCR5Δ32)* mutation [58,59]. Due to few European individuals harboring the natural *CCR5Δ32* mutation, scientists have tried to use genome editing tools to generate induced pluripotent stem cells (iPSCs) homozygous or cluster of differentiation 4 T (CD4^+^ T) cells mimicking the naturally occurring *CCR5Δ32* mutation. As the CRISPR/Cas system has been unveiled to target mammalian cells in vivo, it was immediately developed to treat HIV-1 infection.

##### CRISPR-SpCas9 Targeting Host Factors and HIV-1 Genome

CRISPR-SpCas9 is the first system that has been explored for HIV-1 gene therapy research. Ye et al. reported, utilizing a combination of CRISPR-Cas9 and piggyBac technology, that *CCR5Δ32* mutated iPSCs without off-target modification. These genome edited iPSCs can be differentiated into monocytes/macrophages that demonstrated resistance to CCR5-tropic HIV-1_SF170_ challenge in vitro [60]. Wang et al. showed that TZM.bl cells and a human CD4^+^ T cell line, CEMss-CCR5, transduced with lentiviral plasmids expressing Cas9 and CCR5-sgRNAs have a selective advantage during R5-tropic HIV-1 infection and confer resistance to infection with Bru-Yu2 and infectious molecular clones of transmitted/founder (T/F) HIV-1 isolates such as; pTHRO, pCH040, pWITO, pCH106 and pREJO [61]. Li et al. used a chimeric adenovirus as a vector for the delivery of CRISPR/SpCas9, which resulted in the efficient silencing of CCR5 and, thus, HIV-1 resistance in primary CD4^+^ T cells [62]. Mandal et al. utilizing a dual guide approach generated biallelic CCR5 deletion in primary human CD34^+^ Hematopoietic Stem and Progenitor Cells (HSPCs) and CD4^+^ T cells. The CCR5 modified HSPCs retained multilineage capabilities to differentiate into effector cells: CD19^+^ lymphoid cells and CD11b^+^ myeloid cells. They also confirmed in vivo differentiation of HSPCs using NOD-*Prkdc*^Scid^-*IL2rg*^null^ (NSG) recipients [63]. Xu et al. have disrupted CCR5 in human CD34^+^ HSPCs using SpCas9 and selected sgRNA targeting the Δ32 region. These CCR5 modified HSPCs have differentiation activity in vitro and can resist HIV-1 in NOD*scid Il2rg**^−^***^/^***^−^*** mice (NPG) mice, bringing CRISPR-Cas mediated gene therapy closer to clinical treatment [64]. These studies demonstrate that CRISPR/Cas9 has broad applicability for CCR5 targeted hematopoietic cell based HIV-1 gene therapy. However, aside from the primary receptor CD4, CCR5 is one of the co-receptors for X5 tropic strains of HIV-1 entry into target cells. C-X-C chemokine receptor type 4 (CXCR4) is another co-receptor exploited by X4 tropic strains of HIV-1 [65,66] and should be considered as another important target for genome editing against HIV-1 infection because R5-tropic HIV-1, in the late stages of AIDS, can utilize CXCR4 as an alternative co-receptor to enter new cells and cause faster disease progression [67,68]. In order to address this possibility, we designed two sgRNAs that induce the first generation of CRISPR/SpCas9 to disrupt *CXCR4* in primary human CD4^+^ T cells and generate HIV-1 resistance cells without off-target effects [69]. Thereafter, Hultquist et al. programmed the *CXCR4* or *CCR5* genome in primary CD4^+^ T cells by electroporation of CRISPR/SpCas9 ribonucleoproteins and sgRNA and conferred these cells to a tropism-dependent HIV resistant phenotype [70]. In addition, simultaneous knockout of *CXCR4* and *CCR5*, using one *CXCR4/CCR5* combination sgRNA and CRISPR-SpCas9, was assessed in primary CD4^+^ T cells and showed an inhibition of dual tropic HIV-1 infection [71]. Our group combined one *CCR5* sgRNA together with each of two *CXCR4* sgRNAs in one vector, which induced SpCas9 and disrupted *CXCR4* and *CCR5* simultaneously in primary CD4^+^ T cells. These genome-edited cells have a selective advantage during CCR5-tropic or/and CXCR4-tropic HIV-1 infection and the lenti-X4R5-Cas9 vectors did not have non-specific cleavage or cytotoxicity after both CXCR4 and CCR5 disruption, which showed a specific targeting of HIV-1 receptors [72].

The latent provirus integrated in the genome of Jurkat cells, microglia cells, monocytic cells and HeLa cells can be removed by CRISPR/SpCas9 [73,74,75]. Liao et al. reported that CRISPR/SpCas9 and gRNA targeting the integrated HIV-1 genomic DNA caused resistance to incoming HIV-1 infection in primary cells [76]. To complete the life cycle of HIV-1, some host factors are essential for HIV-1 replication, packaging and budding, thus, knockout of these necessary auxiliary genes is another alternative strategy to block HIV-1 infection. It should be noted that loss of these genes may have no effect on cellular function. Previous studies have found several host dependency factors for HIV-1 such as Lens epithelium-derived growth factor (LEDGF/p75) [77,78], PC4 and SFRS1 Interacting Protein 1 (PSIP1) [79], Transportin-3 (TNPO3) [80], nucleoporin 358 (Nup358) [81], Cleavage and polyadenylation specificity factor subunit 6 (CPSF6) [82] and Activated Leukocyte Cell Adhesion Molecule (ALCAM) [83]. Through electroporation of CRISPR/Cas9 ribonucleoproteins (RNPs) in primary CD4^+^ T cells, Hultquist et al. successfully knocked-out LEDGF or TNPO3, causing a tropism-independent reduction in HIV-1 infection [70]. Recent studies using genome-wide screening approaches have unveiled large numbers of host factors that may be essential for HIV-1 replication and thus provide potential therapeutic targets using the CRISPR-Cas system [80,83,84,85].

##### CRISPR-SaCas9 Targeting Host Factors and HIV-1 Genome

Since the newly identified SaCas9 is only about 3.2 kb, together with sgRNA, it is an ideal cassette to be delivered by an AAV vehicle for gene therapy. Compared to lentivirus and adenovirus, AAV capsids can package less than 4.7 kb of single-stranded DNA [86,87]. In addition, AAVs attract considerable interest in gene therapy because of many advantages, such as low toxicity, lack of pathogenicity, sustained gene expression, safe, efficient delivery and broad serotype or tropism range [22]. However, re-administration with the same AAV serotype causes immune responses and reduced efficacy of AAV delivery and AAV-based expression [21,22]. Compared to other AAV subtype vectors, only AAV6 achieved efficient gene editing in transduced primary T cells and CD34^+^ hematopoietic cells [88,89,90]. We have designed 12 sgRNAs to induce SaCas9 targeting *CXCR4* in HEK293T cell lines and chose two sgRNAs/Sacas9 packaged in AAV6 to transduce primary CD4^+^ T cells, these *CXCR4* edited cells demonstrated X4 tropic HIV-1 resistance [91]. We also used AAV6-sgRNA/SaCas9 to successfully target *CCR5* in primary CD4^+^ T cells and CD45^+^ HSCs in vitro and the *CCR5* edited CD4^+^ T cells showed HIV-1 resistance in NSG mice (paper submitted). Besides, we (manuscript is under revision) and other groups also showed AAV-mediated SaCas9/sgRNA could be used to excise the integrated HIV-1 genome in cultured progenitor/neural stem cells from transgenic mice and in C11 latent cell lines [23,92].

##### CRISPRa Targeting HIV-1 LTR and Host Factors

To date, highly active antiretroviral therapy (HAART) is the global strategy for treatment of HIV-1 infected patients, however, it only represses HIV-1 replication to an undetectable level and is not able to clear the virus from cells. After cession of antiretroviral drugs, HIV will rebound because of the persistence of a viral reservoir, which forms a latent silent provirus [93,94]. Although the provirus can bypass host immune system surveillance, the new viruses generated by activation of quiescent provirus can be repressed by HAART and the virus producing cells can be recognized and cleared by host immune apparatus. Therefore, this so-called “shock and kill” method, in theory, can cure AIDS and draws much attention because of the eradication of HIV infected CD4^+^ T cells [95,96]. Based on these findings, CRISPRa is utilized to reactivate and induce the expression of the quiescent provirus and then this “shock” would activate host immune responses to “kill” the HIV infected cells. Zhang et al. confirmed this proof-of-concept therapeutic approach by using dCas9-SAM system and sgRNAs targeted to the HIV-1 LTR promoter in HIV-1 latent cell lines; TZM-b1, J-Lat, 2D10, 3C9, E4 and CHME5 microglial cell lines [97]. In addition, Sheena et al. have identified a “hotspot” site for sgRNA binding and dCas9-VP64 activation within the viral enhancer sequence in the LTR promoter of HIV-1 [98]. Furthermore, Prajit et al. also reported a dCas9-VP64 SAM complex (containing both dCas9-VP64 and MS2-p65-HSF1) and dCas9-p300 induced HIV-1 expression in cell-based models of latency by targeting to different LTR regions [99]. Ji et al. used single sgRNA targeted to the HIV-1 LTR and dCas9-SunTag-VP64, to effectively reactivate latent provirus transcription in latently infected human T-cell lines such as C11 and A10.6 [100]. As we mentioned above, HIV-1 relies on several host proteins to fulfill efficient virus propagation, the strategy of using CRISPR-Cas mediated knock out of the essential cellular genes can block HIV-1 replication. On the other hand, human cells have developed a defense system to deter key steps of the virus life-cycle, thereby restrict viral replication. These restriction factors can be activated by CRISPRa and function in anti-HIV defense. Until now, several potent restriction factors have been confirmed to effectively inhibit HIV replication. Tripartite motif 5-alpha (TRIM5α) is a cytoplasmic protein that interacts with incoming retroviral capsids (CA) and blocks virus replication in susceptible cell lines and CD34^+^ cell-derived macrophages by a species-specific manner [101,102,103]. A TRIM5αhu R332G/R335G mutant showed strong restriction against HIV-1 infection in a Sup-T1 T-cell line [104]. Martha et al. generated an artificial anti-HIV cassette, hT5Cyp, by fusing the apex of the hTRIM5α PRYSPRY domain with human cyclophilin A (CypA) to mimic the anti-retrovirus function of New World monkeys of the genus Aotus [105]. Apolipoprotein B mRNA-editing enzyme, catalytic polypeptide (APOBEC) proteins are a group of polynucleotide cytidine deaminases that cause the deamination of the cytosine base of either RNA or DNA, thus converting cytidine (C) to uridine (U). The antiviral activity of APOBEC3G is species-specific and can be counteracted by HIV-1 Vif [106]. APOBEC3G with D128K mutation should block antagonism by Vif and restore A3G’s cytidine deaminase activity on viral RNA [107,108]. BST-2/Tetherin/CD137/HM1.24 is an interferon induced antiviral host protein and prevents HIV-1 replication through the inhibition of viral particle release [109,110]. Tetherin is counteracted by the HIV-1 Vpu protein and substitution of a single amino acid (T45I) in Tetherin confers Vpu-resistance [111]. Sterile Alpha Motif- and HD-domain containing protein 1 (SAMHD-1) is a key Aicardi–Goutières syndrome related gene whose function as a restriction factor inhibits lentivirus infection in myeloid cells. This restriction can be counteracted by Vpx through Vpx-induced proteasomal degradation of SAMHD1 [112,113]. SAMHD1 is a dGTP-dependent deoxynucleoside triphosphohydrolase that reduces intracellular dNTPs below the levels required for HIV-1 reverse transcription, thereby blocking HIV-1 infection in resting CD4^+^ T-cells [114,115,116,117]. Myxovirus resistance protein 2 (Mx2) is an interferon induced inhibitor of HIV-1 infection that prevents capsid-dependent nuclear import of subviral complexes in the late stage during infection [118,119]. Voit et al. found that the combination of CCR5 disruption with restriction factor over-expression has significant protective effects against both R5-tropic and X4-tropic HIV-1 infection. They designed targeting vectors by combinations of APOBEC3G D128K, hrhTRIM5α and Rev M10 (“CCR5-APO-hrh”, “CCR5-hrh-triple”, “CCR5-Rev-APO”, “CCR5-Rev-hrh”) to block HIV-1 infection at multiple stages, thus providing a roadmap for CRISPR target application [120]. For the application of CRISPRa targeting HIV restriction factors, only one paper showed that the SAM system with both single sgRNA and combination of two sgRNAs activates endogenous APOBEC3G (A3G) and APOBEC3B (A3B) to inhibit HIV-1 (Δvif) infection by convert dC-to-dU editing of HIV genome [121]. However, activation of other host genetic regulators by CRISPRa need further investigation. Recently, Serinc5/Nef pair of HIV-1 restriction factor/viral accessory protein that have been recently discovered and can also be potentially targeted by CRISPRa [122,123].

#### 1.3.2. Hepatitis B Virus (HBV)

HBV infection and its related disease remain a serious threat to human health. HBV contains a relaxed circular (RC) DNA genome, which can be converted to covalently closed circular (ccc) DNA by cellular enzymes in infected hepatocytes. The ccc DNA transcribes four viral RNAs including pregenomic RNA (pgRNA), preS, S and X. The pgRNA serves as a template and reverse transcript to genomic DNA, or, together with other sub-genomic RNAs, such as mRNA, produces the core (C), polymerase (P), surface (S), viral M, glycoproteins, HBeAg and hepatitis B virus X (HBx) proteins. Based on the principle of CRISPR/Cas9 cleavage, HBV cccDNA is the ideal target of CRISPR/Cas9 gene editing [124,125]. Several studies have utilized SpCas9 to edit conserved open reading frames (ORFs) in the HBV cccDNA to eradicate virus in cells or mouse models [126,127,128,129,130,131,132,133,134,135,136,137,138,139,140]. However, there is no report of using the CRISPR-Cas system in HBV infected primary cells. Which kind of CRISPR-Cas system will more efficiently edit the HBV genome also needs further investigation and comparison. Aside from virus genome editing, host factors also could be a target for prevention of HBV infection. The first target to be considered should be the identified receptor, bile acid pump sodium taurocholate cotransporting polypeptide (NTCP) [141]. Inhibition of NTCP protein expression prevents HBV replication, suggesting that knockout of NTCP by the CRISPR-Cas system may be a strategy to block HBV infection [135,141,142,143,144,145,146,147]. Other key host factors involved in HBV replication can also be considered as targets. For example, heat stress cognate 70 (Hsc70) is an essential protein for HBV replication but not necessary for cell survival. Thus, CRISPR-Cas editing Hsc70 may contribute to the inhibition of HBV replication [148,149,150]. The heat shock protein Hsp90 is known as a pivotal component of several signaling pathways and has now been reported as a necessary host factor for hepatitis B virus replication [151,152,153]. During the HBV life cycle, Tyrosyl-DNA-phosphodiesterase 2 (TDP2) has been identified to specifically break a tyrosine-DNA bond in HBV (DHBV) RC-DNA, releasing the viral P protein and helps conversion of RC-DNA into cccDNA. Thus, knock-down of TDP2 in human cells dramatically decelerated the conversion of RC-DNA to cccDNA [154,155]. Researchers have also identified that γ^2^-adaptin, a putative member of the clathrin adaptor proteins involve in protein sorting and trafficking, binds to L protein and, together with pre-S domain, perform crucial functions by mediating the attachment of HBV to liver cells [156,157]. BST-2/tetherin is an interferon-inducible antiviral factor that blocks the release of diverse enveloped viruses, including HBV [158,159,160]. Altogether, these host factors could be targeted by the CRISPR-Cas system to treat HBV related disease. Furthermore, the HBV regulatory IncRNA and pgRNA, as well as sub-genomic RNA, should also be considered for targeting by an RNA editing system, such as FnCas9 and CRISPR-Cas13.

#### 1.3.3. Human Papillomavirus (HPV)

According to the WHO report, although two effective Human papillomavirus (HPV) vaccines are now available, HPV related cervical cancer remains the second most common cancer in women worldwide, classified by age-standardized incidence rate (ASR) (http://www.who.int/immunization/topics/hpv/en/). HPV can also induce other types of anogenital cancer, head and neck cancers and genital warts in both men and women. The dsDNA genome of HPV may integrate into the host cell chromosome, which makes its genome an ideal target for CRISPR-Cas editing. Among the more than 200 identified genotypes of HPV [161], the most commonly high-risk HPV types are HPV16 and HPV18, which are detected in cervical and penile cancers frequently [162]. HPV6 and HPV11 are the low-risk HPV types, which are associated with anogenital warts and laryngeal papillomatosis [163]. HPV-related tumorigenesis has been related to the deregulated expression of two oncogenic proteins HPV E6 and E7, which are important for both malignant transformation and maintenance of the malignant phenotype of cervical cancer [164,165]. HPV invades host cells and expression of E6 and E7 can induce degradation of tumor-suppressive protein p53 (E6) and inactivation of retinoblastoma (Rb) protein (E7), thus blocking the cell cycle [165]. Therefore, these two viral oncogenes become the main targets to combat HPV infections.

CRISPR/Cas9 technology has been used in HPV-associated cancer. Kennedy et al. designed 18-special sgRNAs to target the ORF of E6 and E7 that integrated into the human SiHa and HeLa cervical carcinoma cell line genomes [166]. RNA-guided CRISPR/SpCas9 was able to inactivate and cleave both the E6 and E7 gene in HPV-18- transformed SiHa cells and HPV-16-transformed HeLa cells respectively [166]. Zhen et al. also used CRISPR/SpCas9 system to target E6 and E7 leading to the enhanced expression of p53 and Rb, accelerating HPV16-infected SiHa cells apoptosis [167]. Furthermore, E6/E7-gRNAs-modified SiHa cells were introduced into BALB/C nude mice to reduce the tumor size compared to the control [167]. Many host cell restriction factors that limit transcription and replication of HPV such as Sp100, miR-145, p56, Gamma-interferon-inducible protein 16 (IFI16), CCAAT/Enhancer Binding Protein Beta (C/EBPβ), p53, Epidermodysplasia verruciformis (EVERs), APOBEC, Fanconi Anemia Complementation Group D2 (FANCD2) et al. could be activated by CRISPRa to block HPV infection [168]. The recent functional studies also provide other host targets for HPV gene therapy such as Sirtuin 1 (SIRT1) [169], Bromodomain-containing protein (Brd4) [170], CXCL12/CXCR4 [171], Kruppel Like Factor 4/13 (KLF4/13) [172,173], Origin recognition complex subunit 2 (ORC2) [174] and microRNA-146a [175].

#### 1.3.4. Epstein–Barr Virus (EBV)

Epstein–Barr virus (EBV) belongs to the gamma 1 herpes virus family and contains a linear dsDNA. EBV infection causes lifelong persistent infections in more than 90% of adult people [176]. Although EBV does not induce disease in the healthy carrier, it can cause infectious mononucleosis and is strongly associated with many lymphoid and epithelial malignancies such as post-transplant lymphoproliferative disorders (PTLD), gastric carcinoma, Burkitt’s lymphoma, Hodgkin’s lymphoma and nasopharyngeal carcinoma [177,178,179,180]. The majority of EBV-associated diseases are induced by its latency and the CRISPR/Cas9 system has been used to target herpesviruses, especially in the treatment of EBV. Wang et al. designed seven guide RNAs targeting six different regions in the EBV genome for different editing purposes and then transfected with a Burkitt’s lymphoma patient-derived B cell line. The targeting CRISPR-SpCas9/sgRNAs reduces episomal EBV genome content in the infected cells, leading to a dramatic cell proliferation arrest and concomitant decrease in viral load [181]. Subsequently, Yuan et al. designed two sgRNAs targeting a 558 bp region in the promoter region of *BART* (*Bam*HI A rightward transcript), a major viral transcript derived from latently infected cells that encodes the viral microRNAs (miRNAs). They observed gene disruption in nasopharyngeal carcinoma C666-1 cells latently infected with EBV, which indicated potential treatment of EBV infection [182]. Furthermore, van Diemen et al. used single sgRNA targeting the EBV nuclear antigen 1 (EBNA1) and the origin of replication region by lentivirus mediated CRISPR/Cas9 in Burkitt’s lymphoma Akata-Bx1 cells could deplete 40–60% of EBV genome, whereas targeting *EBNA1* with two different sgRNAs simultaneously made the efficiency as high as 90% [183]. Recently, a CRISPR/Cas9 screen was used to explore EBV transformed B cell host dependency factors [184]. In order to find out host dependency factors resulting from EBV+ lymphoblastoid cells, researchers conducted parallel genome-wide CRISPR/Cas9 loss-of-function screens in BL and lymphoblastoid cell lines (LCLs) [184]. This systematic approach identified key mechanisms that EBV oncoproteins activated the PI3K/AKT pathway and evaded tumor suppressor responses [184]. EBV-induced BATF/IRF4 were essential for MYC induction and BIM suppression in LCLs and EBV super-enhancer-targeted IRF2 could protect LCLs form Blimp1-mediated tumor suppression [184]. Aside from EBV, the CRISPR/Cas9 system had also been used in other herpesviruses such as HSV-1 [183,185,186] and Human cytomegalovirus (HCMV) [183] genome editing. Therefore, the CRISPR/Cas9 system is a promising technology for the depletion of EBV in vivo, yet further studies must be conducted to test its feasibility.

### 1.4. CRISPR and Human RNA Viruses

As mentioned above, much research has reported the application of CRISPR/Cas9 system in pathogenic human viruses that contain a dsDNA intermediate in their infection cycle. However, for the RNA virus genome, canonical CRISPR/Cas9 cannot perform its editing function. Among all CRISPR-Cas system, the Cpf1 and C2c2/Cas13 could be designed for RNA virus genome targeting. Atypical Cas9 from *F. novicida* (FnCas9) was reported to target an endogenously transcribed mRNA and regulate gene expression [37]. Therefore, except for RNA mediated DNA editing systems targeting host factors—especially specific receptors that control RNA virus inhibition—these RNA-induced RNA targeting apparatus provide the possibility to edit both human RNA virus genomes or the mRNA of host factors.

#### 1.4.1. Hepatitis C Virus (HCV)

HCV has a positive-strand RNA genome that consists of a 5′-non-coding region (NCR), an ORF and a 3′-NCR [187]. It is one of the main pathogens causing chronic hepatitis, liver cirrhosis and hepatocellular carcinomas. People infected with HCV can become chronic carriers [188]. HCV is not only associated with liver diseases but often along with HIV infection [189]. Price et al. utilized FnCas9 to target the 5′-and 3′-UTR of the HCV genomic +ssRNA in human hepatocellular carcinoma cells (Huh-7.5). They observed virus inhibition due to blockade of viral RNA translation and viral replication machineries, rather than genomic RNA breakage [38]. The inhibition function of FnCas9 is the same as RNAi or CRISPRi that impede mRNA expression. Since the CRISPR-Cas system was developed for mammalian cell genome editing, it is programmed to screen host factors for regulation of various virus life cycle. One of the applications of the CRISPR/Cas9 library is the usage of the NIrD system to identify essential genes for HCV. Ren et al. found three genes Cluster of Differentiation 81 (CD81), Claudin 1 (CLDN1) and Occludin (OCLN) which were necessary for both the cell-to-cell transmission and the cell-free entry of HCV infection [190]. Alternatively, the CRISPR/Cas9 library should be designed to screen more regulatory targets such as IncRNA or miRNA for HCV life cycle [191].

#### 1.4.2. Other RNA Virus: Dengue Virus (DENV) and Zika Virus (ZIKV), West Nile Virus (WNV), Influenza Virus, Ebola Virus (EBOV), SARS-CoV, MERS-CoV

The outbreak of flaviviruses, ZIKV and DENV, or other non-flaviviruses such as WNV, EBOV, SARS-CoV, MERS-CoV and Influenza virus, often cause acute disease and induce apprehensive panic in the world. Effective drugs and alternative therapies that inhibit the spread of acute viral infection is the hot topic in research. The basic prerequisite of blocking these pandemics is to understand the mechanism of virus-host interactions. Researchers have spent much effort to identify essential host factors for these rapidly emerging pathogens [192,193]. A genome-wide CRISPR/Cas9-based knockout screen to identify genes essential for flavivirus infection, Zhang et al. confirmed nine human genes contribute to replication of Flaviviridae family members including, WNV, DENV, ZIKV, yellow fever virus, Japanese encephalitis virus and HCV. The most important target is a subset of endoplasmic reticulum-associated signal peptidase complex (SPCS) proteins which mediate cleavage of the flavivirus structural proteins (prM and E) and release of viral particles [194]. At the same time, through a pooled CRISPR genetic screening strategy, Marceau et al. identified the endoplasmic-reticulum (ER)-associated multi-protein complexes involved in DENV and ZIKV replication. They also found the interaction of oligosaccharyltransferase (OST) complex with DENV non-structural proteins NS1 and micro RNA-122 as well as micro RNA-DiGeorge syndrome chromosomal region 8 (DGCR8) are important for HCV replication [195]. In addition, Savidis et al. combined RNAi and CRISPR/Cas9 approaches to verify that AXL Receptor Tyrosine Kinase (AXL), RAB5C, RABGEF, N-Deacetylase and N-Sulfotransferase 1 (NDST1), Exostosin Glycosyltransferase 1 (EXT1) and endoplasmic reticulum (ER) membrane complex (EMC) are important in DENV and ZIKV infection at different life cycle stages [196]. Moreover, Gootenberg et al. developed a detection platform named Specific High-Sensitivity Enzymatic Reporter UnLOCKing (SHERLOCK) to discriminate specific strains of DENV and ZIKV by using C2c2/Cas13a and isothermal amplification [197]. Ma et al. identified seven endoplasmic reticulum-associated protein degradation (ERAD) pathway related genes containing EMC2, EMC3, ER-associated degradation (ERAD) E3 Ligase Adaptor Subunit (SEL1L), Derlin 2 (DERL2), UBE2G2, UBE2J1 and ERAD-associated E3 ubiquitin-protein ligase HRD1, that are important but not essential to WNV replication [198]. Heaton et al. utilized CRISPRa system to identify Influenza virus restriction factors on a genomic scale level and found Beta-1,4-N-Acetyl-Galactosaminyltransferase 2 (B4GALNT2) can inhibit several Influenza virus strains binding to α 2,3 linked sialic acid receptor to abolish infection [199].

The 2013–2016 Ebola virus (EBOV) disease (EVD) epidemic in West Africa has attracted much attention in the world. According to statistical analysis of WHO, EBOV caused 28,646 cases and 11,323 deaths, mainly in Guinea, Sierra Leone and Liberia [200]. The EBOV contains a 19 kb single-stranded RNA genome which encodes seven structural proteins: nucleoprotein (NP), polymerase cofactor (VP35), (VP40), GP, transcription activator (VP30), VP24 and RNA-dependent RNA polymerase (L) [201]. In order to control EVD and EBOV transmission, efforts -have been made by scientists to identify effective vaccines. Two candidates—chimpanzee adenovirus 3 vaccine (ChAd3-EBO-Z) and the recombinant vesicular stomatitis virus vaccine (rVSVΔG-ZEBOV-GP) were evaluated by a phase 2 clinical trials but have not expanded to phase 3 because of the number of EVD patients declined [202]. Through the screening of a library of small molecules, a novel benzylpiperazine adamantane diamide which targets the endosomal membrane protein Niemann–Pick C1 (NPC1), was identified to inhibit EboV infection in cells [203]. They further confirmed that NPC1 is the receptor of EBOV [204]. Carette et al. have not only found the NPC1 receptor for EBOV but also uncovered 67 host factors involved in the filoviruses entry process [205]. Younan and Kondratowicz both revealed that T-cell immunoglobulin and mucin domain-containing protein 1 (Tim-1) can interact with virion-associated phosphatidylserine to promote EBOV infection and act as another receptor [206,207]. All these above factors could be considered as potential gene editing targets for EBOV treatment.

In 2003, severe acute respiratory syndrome (SARS) spread rapidly through the world and a new coronavirus (SARS-CoV) was identified as the disease pathogen. It harbors a large genomic RNA with 2 large ORFs, 1a and 1b, encoding viral replicase/transcriptase, structural and virus specific accessory proteins [208,209]. In the same year, Li et al. found angiotensin-converting enzyme 2 (ACE2) is the functional receptor for SARS-CoV in cells [210]. Further proof of ACE2 function in vivo was confirmed by two groups [211,212]. Huang et al. have indicated that Interferon-inducible transmembrane proteins (IFITM) also play a restriction role in Filoviruses, SARS-CoV and Influenza virus entry, thus providing alternative targets for gene editing [213]. The 2013 outbreak of Middle East respiratory syndrome coronavirus (MERS-CoV), unlike SARS-CoV, use CD26 (also named as dipeptidyl peptidase 4, DPP4) as receptor binding to MERS-CoV spike to mediate membrane fusion [214,215,216]. Other host proteins, such the tetraspanin CD9 was found to form a complex with CD26 for early, efficient MERS-CoV entry [217]. More and more host factors should be identified using a genome library screen by CRISPR and offers alternative gene targets for these re-emerging viruses.

### 1.5. CRISPR and Plant Viruses

Geminivirus is a circular, single-stranded DNA (ssDNA) virus that is a serious threat to plants including vegetables, crops and fruit [218,219]. An RNA-based interference strategy has been used to achieve geminivirus resistance but this transgenic approach has limitations, including double stranded RNA induced sequence-specific DNA methylation and incomplete elimination of virus [220]. Because of the large family of Geminiviridae [221], controlling the infection of geminivirus becomes inefficient and expensive. However, in recent years, the CRISPR/Cas system mediated virus resistance has been used not only in humans but also in plants. By directly editing the viral genes or disrupting the host factors required for virus replication, Geminiviruses could be effectively resisted in plants [222,223,224,225].

Baltes and colleagues used the CRISPR/SpCas9 system in transgenic *Nicotiana benthamiana* plants to target the replication initiator protein gene of bean yellow dwarf virus (BeYDV), which introduced virus resistance [225]. Ji et al. showed that CRISPR/Cas9 system could perform high viral resistance in *N. benthamiana* against geminivirus and beet severe curly top virus (BSCTV). They also adjusted Cas9 and gRNA expression with the levels of virus suppression and demonstrated that higher expression levels of Cas9 resulted in no obvious viral symptoms [222]. Ali et al. also programmed the CRISPR/SpCas9 system to target the viral Rep, coat proteins encoded genes and the conserved intergenic region (IR) in *N. benthamiana* plants against the Tomato yellow leaf curl virus (TYLCV), Beet curly top virus (BCTV) and Merremia mosaic virus (MeMV) for virus resistance. Their group also targeted the conserved IR region in *N. benthamiana* plants resulting in the broad spectrum high virus interference against multiple viruses without viral variant escapes, thus this strategy provided a feasible method for broad and durable resistance in *N. benthamiana* plants [224].

## 2. Challenge of Using CRISPR-Cas System Targets Host Factors to Protest against Virus Infection in Humans

### 2.1. Virus Mutation and Host Immune System

As various viruses can escape host immune monitoring by frequent genome mutation during replication, it is not efficient using one gRNA to target the virus genome [132,226]. In addition, virus can evolve to utilize alternative host receptors to enter cells, which leads to consideration of targeting more than one host gene at a time when using the facile and versatile Cas9/gRNA technology platform [72]. In order to address this issue, multiple sgRNAs should be combined to target both viral genome and host factors. Nowadays, it is possible to isolate DNA and determine viral sequences in individual patients. One day this may lead to designed, personalized sgRNAs to induce a CRISPR-Cas system targeting sequenced viral genomes quickly, easily and efficiently. There is no data is available concerning how the mammalian immune system responds to CRISPR-Cas apparatus. Whether various effects from proteins such as SpCas9, Sa-Cas9, dCas9 and CPF1 induce cross-reactive antibodies or T cell responses is unknown. Are there any CRISPR-Cas regulators in host cells? How to identify these factors?

### 2.2. Off Target and Side Effect

The CRISPR/Cas system represents a powerful tool for genome engineering in antivirus gene therapy; however, the potential off-target cleavage is always a serious hurdle for future clinical applications. In order to select the most highly specific and efficient sgRNAs, we should align target sequences to the human genome to search for all the potential off-target sites using an online tool. Experimental confirmation of these off-target effects is always a fundamental prerequisite for further therapeutic treatment. Sometimes the whole genome sequence is needed before application to patients. For a specific target, not every sgRNA has the effect to induce CRISPR-Cas cleavage or activation, consequently, one should choose the sgRNA which has the highest efficacy and lowest off target effect. For the knockout of genes in host cells by the CRISPR-Cas system, the lack of target gene should not affect normal physiological function in cells. Concerning the forced expression of endogenous genes that restrict virus replication, the overloaded protein should not induce cytotoxic effects in cells. In terms of proving alternative targets, more and more host factors must be identified that are important for regulation of the virus lifecycle.

### 2.3. Delivery Method

For the CRISPR-Cas system, as a large cassette of DNA/protein complex, the safety and efficiency of delivering reagents is another technical concern. The best method of delivery is transient expression of Cas protein:gRNAs or Cas mRNA:gRNA to cells. However, this method often relies on a highly technical electroporation machine and a large quantity of primary cells due to high voltage induced cell death. In addition, the in vitro expression of Cas protein and transcription of gRNA or synthesis of gRNA costs labor, work and money. Based on previous reports, the delivery vector for CRISPR-Cas contains adenoviral (AV), lentiviral (LV) and adeno associatedviral (AAV) plasmids. AV is suitable for all kinds of CRISPR-Cas systems because of its large incorporation capacity of the extra DNA fragment. It is easy to employ various engineered packaging cells to generate recombinant AV, however, the financial cost and labor intensive methods make it difficult to generate recombinant AV vector [227]. Moreover, the significant immunogenic effect in clinical usage has been addressed by the engineering of second and third generation of AV vectors [228]. On the other hand, lentiviral vector mediated CRISPR-Cas delivery has been widely used for gene targeting in both dividing and non-dividing cells, however, it still has a high risk of induction of tumorigenesis due to integration of host genome. An integrase-defective lentivirus could be a valuable choice because of the transient expression. In the case of AAV, because the packaging size is less than 4.7 kb of single-stranded DNA, it leaves little space for inserting other genetic elements when infusing SpCas9. The SaCas9 and CPF1 can be packaged into the AAV genome together with a sgRNA gene expression cassette. AAV-mediated SaCas9/sgRNA has shown to excise the integrated HIV-1 genome in vivo [23,92]. Using AAV as a gene therapy vector has many advantages, such as low toxicity, sustained gene expression, safe and efficient delivery [22]. AAV6 was reported to transduce primary T cells and CD34^+^ hematopoietic cells [88,89,90]. In addition, AAV8 and 9 have been used to deliver Cas9/gRNAs to modify disease-causing mutations in mouse models of Duchenne muscular dystrophy, suggesting the *in vivo* potential of Cas9 delivery by AAV. Because of the crystal structure of Cas have been explored, the subdomains of Cas could be assembled separately into AAV and reconstituted in cells. In order to deliver the CRISPR-Cas system to function in specific cells or organs, one should choose suitable inducible promoters to regulate Cas/gRNA expression in the target cells or organs.

### 2.4. Clinical Trials

A decade ago, the “Berlin patient” with acute myeloid leukemia (AML) and HIV-1 infection accepted a bone-marrow transplant from a natural *CCR5 Δ 32* mutation donor for leukemia therapy, which also blocked his HIV-1 infection without further ART [58,59]. This case opens a new era for cell-based gene therapy, leading to several clinical trials of HIV-infected individuals using ZFN-modified autologous T cells and HSPCs [229]. However, the CRISPR-Cas based clinical test for treatment of AIDS or other virus related disease needs further evaluation. Since the CRISPR-Cas system has emerged as a new gene editing technology and various Cas analogues have been developed, it is plausible to compare both the advantages and drawbacks of every method and select a suitable one to translate from the bench to the bedside. At the same time, researchers also should try to find more and more host factors involved in the virus life cycle and offer more targets for CRISPR-Cas editing.

## 3. Conclusions and Future Directions

CRISPR-Cas based targeting of host genes represents an alternative solution for virus related disease treatment for future applications. Weatherley et al. predicted an AAV-SaCRISPR-Cas9 mediated ex vivo gene editing of TRIM5α in hematopoietic stem cells (HSCs) to cure HIV [230]. As an antiviral strategy, gene therapy shows many promising clinical results [59,231], however, financial investment and research efforts must be made for the enhancement of this technology. (1) Discovery of additional, new, CRISPR-Cas systems and improvements of these newly identified systems will yield high gene editing efficiencies and low off target cleavage; (2) Explore the mechanism of virus escape from long term CRISPR-Cas editing in cells; (3) Demonstrate the host immune response to various CRISPR-Cas system and regulators involved in the DNA targeting process; (4) Off target screening and related test method development; (5) Virus life cycle dependent host target selection and side effects of these gene knock in/out or activation; (6) Development of new CRISPR-Cas delivery vehicles and assembly of functional cassettes; (7) Preclinical tests in virus infected animal models.

## Figures and Tables

**Table 1:** Comparison of different CRISPR-Cas systems in eukaryotic cells.

CRISPR-Cas		DNA Cassette: Size	PAMs	Function	Targeting Virus	Virus Related Host Factor	Delivery Vehicle	Pros/Cons
CRISPR-SpCas9		Sp-Cas9-Flag-T2A-Puro:4641 bp	5′-NGG-3′	single-RNA-mediated DNA endonuclease	HIV, HBV, HPV, EBV, ZIKV, DENV	CCR5, CXCR4, LEDGF, TNPO3, BART, EBNA1, CLDN1, OCLN, CD81, AXL, EMC	Adenoviral vector Lentiviral vector Retroviral vector	Frequently used/Large protein size
CRISPRi		Flag-NLS-dCas9-NLS-KRAB-T2A-Puro:5145 bp	5′-NGG-3′	single-RNA-mediated inhibition of mRNA transcription	-	-	Lentiviral vector Retroviral vector	Multiplex gene transcription inhibition/Large protein size, less application
CRISPRa	dCas9-VP64 and MS2–p65–HSF1	dCas9-VP64-Blast:4872 bp MS2-p65-HSF1-Hygro:2520 bp	5′-NGG-3′	single-RNA-mediated activation of mRNA transcription	HIV	IFNγ, APOBEC3G (A3G), APOBEC3G (A3G)	Lentiviral vector	Multiplex gene activation/Relative low efficiency, Large protein size, less application
	scFv-GCN4-sfGFP-VP64 and dCas9-24xGCN4-v4	scFv-GCN4-sfGFP-VP64-GB1-NLS:2031 bp NLS-dCas9-24xGCN4_v4-NLS-P2A-BFP:6906 bp						Single-molecule imaging, transcriptional activation/Super large in size, less application
CRISPR-SaCas9		NLS-SaCas9-NLS-3xHA:3411 bp	5′-NNGRRT-3′	single-RNA-mediated DNA endonuclease	HIV	CCR5, CXCR4	AAV vector Lentiviral vector	Small in size/Relatively strict PAM, less application
Cpf1		AsCpf1:4056 bp LbCpf1:3819 bp FnCpf1:3900 bp	5′-TTTN-3′	single-crRNA-mediated DNA Endonuclease/RNase activity	-	-	pcDNA3.1 vector	a single and short crRNA, cohesive ends, Multiplex gene editing with tandemly arrayed crRNA/less application in cells
FnCas9		FnCas9:4887 bp	5′-NGG-3′	single-RNA-mediated PAM-independent inhibiting of translation of target RNA	HCV	-	pcDNA3.3 vector	RNA targeting, Less restrictive PAM/Large in size, less application
C2c1/3		AacC2c1:3387 bp	5′-TTN-3′	dual-RNA-guided DNA endonuclease	-	-	No mammalian expression vector	Small in size/requires a 111-nt sgRNA containing crRNA and tracrRNA, lower cleavage activity, no application in cells
C2c2/Cas13		LwCas13a-msfGFP-2A-Blast:4869 bp	depends on a non-G 3′ protospacer-flanking site (PFS)	single-effector endoRNase mediating ssRNA cleavage with a single crRNA guide	ZIKV, DENV	-	pFUGW vector	RNA targeting, nucleic acid detection/Large in size, less application

**Table 2:** Potential anti-viral host factors for targeting by CRISPR-Cas system.

Virus	Genome	CRISPR-Cas for Genetic Targeting	Dependent or Positive Regulators	Restriction Factors
HIV	+ssRNA	CRISPR-SpCas9, CRISPRa CRISPR-SaCas9 (Cpf1, FnCas9, C2c1/3, C2c2/Cas13)	CCR5, CXCR4, LEDGF/p75, TNPO3 (PSIP1, Nup358, CPSF6, ALCAM)	APOBEC3G, APOBEC3B, IFNγ (TRIM5α, BST-2/Tetherin, SAMHD-1, Mx2, Serinc3/5)
HBV	relaxed circular (RC) DNA	CRISPR-SpCas9 (CRISPR-SaCas9, Cpf1, FnCas9, C2c1/3, C2c2/Cas13)	(NTCP, Hsc70, Hsp90, TDP2, γ^2^-adaptin)	IFNγ (BST-2/tetherin)
HPV	dsDNA	CRISPR-SpCas9 (CRISPR-SaCas9, Cpf1, FnCas9, C2c1/3, C2c2/Cas13)	(SIRT1, Brd4, CXCR4, KLF4/13, ORC2)	(Sp100, miR-145, p56, IFI16, C/EBPβ, p53, EVERs, APOBEC, FANCD2)
EBV	dsDNA	CRISPR-SpCas9 (CRISPR-SaCas9, Cpf1, FnCas9, C2c1/3, C2c2/Cas13)	IKKβ, HOIP, p52, RBP-Jκ, WDR48, MDM2/4, CTBP1, BCL6,SYK,BTK,BLNK,PTEN, CD19/81, TNFRSF1A, BATF, IRF4, IRF2	(c-CBL)
HCV	+ssRNA	FnCas9 (C2c2/Cas13)	DGCR8, CLDN1, OCLN, CD81	(NLRX1, SMYD3, VAP-C)
ZIKV	+ssRNA	C2c2/Cas13 (FnCas9)	(OST, AXL, RAB5C, RABGEF, NDST1, EXT1, EMC)	-
DENV	+ssRNA	C2c2/Cas13 (FnCas9)	(OST, RAB5C, RABGEF, NDST1, EXT1, EMC)	-
WNV	+ssRNA	(FnCas9, C2c2/Cas13)	(SPCS, EMC2, EMC3, SEL1L, DERL2, UBE2G2, UBE2J1, HRD1)	(HSP70)
IAV	−ssRNA	(FnCas9, C2c2/Cas13)	(IFITM, B4GALNT2, α2,3 linked sialic acid receptor)	(DcR3)
EBOV	+ssRNA	(FnCas9, C2c2/Cas13)	(NPC1, Tim-1)	-
SARS-CoV	+ssRNA	(FnCas9, C2c2/Cas13)	(ACE2, IFITM)	-
MERS-CoV	+ssRNA	(FnCas9, C2c2/Cas13)	(CD26/DDP4, CD9)	-

Brackets indicate that CRISPR-Cas may be used or potential host factors could be targeted in the future. The −ssRNA means negative-sense single-stranded RNA and +ssRNA means positive-sense single-strand RNA.

## References

[1] Brouns S.J., Jore M.M., Lundgren M., Westra E.R., Slijkhuis R.J., Snijders A.P., Dickman M.J., Makarova K.S., Koonin E.V., van der Oost J. (2008). Small CRISPR RNAs guide antiviral defense in prokaryotes. Science.

[2] Garneau J.E., Dupuis M.E., Villion M., Romero D.A., Barrangou R., Boyaval P., Fremaux C., Horvath P., Magadan A.H., Moineau S. (2010). The CRISPR/Cas bacterial immune system cleaves bacteriophage and plasmid DNA. Nature.

[3] Barrangou R., Horvath P. (2017). A decade of discovery: CRISPR functions and applications. Nat. Microbiol..

[4] Barrangou R., Marraffini L.A. (2014). CRISPR-Cas systems: Prokaryotes upgrade to adaptive immunity. Mol. Cell.

[5] Jinek M., Chylinski K., Fonfara I., Hauer M., Doudna J.A., Charpentier E. (2012). A programmable dual-RNA-guided DNA endonuclease in adaptive bacterial immunity. Science.

[6] Cong L., Ran F.A., Cox D., Lin S., Barretto R., Habib N., Hsu P.D., Wu X., Jiang W., Marraffini L.A. (2013). Multiplex genome engineering using CRISPR/Cas systems. Science.

[7] Mali P., Yang L., Esvelt K.M., Aach J., Guell M., DiCarlo J.E., Norville J.E., Church G.M. (2013). RNA-guided human genome engineering via Cas9. Science.

[8] Hsu P.D., Lander E.S., Zhang F. (2014). Development and applications of CRISPR-Cas9 for genome engineering. Cell.

[9] Shalem O., Sanjana N.E., Hartenian E., Shi X., Scott D.A., Mikkelsen T.S., Heckl D., Ebert B.L., Root D.E., Doench J.G. (2014). Genome-scale CRISPR-Cas9 knockout screening in human cells. Science.

[10] Wang T., Wei J.J., Sabatini D.M., Lander E.S. (2014). Genetic screens in human cells using the CRISPR-Cas9 system. Science.

[11] Zhou Y., Zhu S., Cai C., Yuan P., Li C., Huang Y., Wei W. (2014). High-throughput screening of a CRISPR/Cas9 library for functional genomics in human cells. Nature.

[12] Nishimasu H., Ran F.A., Hsu P.D., Konermann S., Shehata S.I., Dohmae N., Ishitani R., Zhang F., Nureki O. (2014). Crystal structure of Cas9 in complex with guide RNA and target DNA. Cell.

[13] Jiang F., Zhou K., Ma L., Gressel S., Doudna J.A. (2015). STRUCTURAL BIOLOGY. A Cas9-guide RNA complex preorganized for target DNA recognition. Science.

[14] Qi L.S., Larson M.H., Gilbert L.A., Doudna J.A., Weissman J.S., Arkin A.P., Lim W.A. (2013). Repurposing CRISPR as an RNA-guided platform for sequence-specific control of gene expression. Cell.

[15] Gilbert L.A., Larson M.H., Morsut L., Liu Z., Brar G.A., Torres S.E., Stern-Ginossar N., Brandman O., Whitehead E.H., Doudna J.A. (2013). CRISPR-mediated modular RNA-guided regulation of transcription in eukaryotes. Cell.

[16] Maeder M.L., Linder S.J., Cascio V.M., Fu Y., Ho Q.H., Joung J.K. (2013). CRISPR RNA-guided activation of endogenous human genes. Nat. Methods.

[17] Perez-Pinera P., Kocak D.D., Vockley C.M., Adler A.F., Kabadi A.M., Polstein L.R., Thakore P.I., Glass K.A., Ousterout D.G., Leong K.W. (2013). RNA-guided gene activation by CRISPR-Cas9-based transcription factors. Nat. Methods.

[18] Konermann S., Brigham M.D., Trevino A.E., Joung J., Abudayyeh O.O., Barcena C., Hsu P.D., Habib N., Gootenberg J.S., Nishimasu H. (2015). Genome-scale transcriptional activation by an engineered CRISPR-Cas9 complex. Nature.

[19] Tanenbaum M.E., Gilbert L.A., Qi L.S., Weissman J.S., Vale R.D. (2014). A protein-tagging system for signal amplification in gene expression and fluorescence imaging. Cell.

[20] Ran F.A., Cong L., Yan W.X., Scott D.A., Gootenberg J.S., Kriz A.J., Zetsche B., Shalem O., Wu X., Makarova K.S. (2015). In vivo genome editing using Staphylococcus aureus Cas9. Nature.

[21] Zaiss A.K., Muruve D.A. (2008). Immunity to adeno-associated virus vectors in animals and humans: A continued challenge. Gene Ther..

[22] Mingozzi F., High K.A. (2013). Immune responses to AAV vectors: Overcoming barriers to successful gene therapy. Blood.

[23] Kaminski R., Bella R., Yin C., Otte J., Ferrante P., Gendelman H.E., Li H., Booze R., Gordon J., Hu W. (2016). Excision of HIV-1 DNA by gene editing: A proof-of-concept in vivo study. Gene Ther..

[24] Kaya H., Mikami M., Endo A., Endo M., Toki S. (2016). Highly specific targeted mutagenesis in plants using Staphylococcus aureus Cas9. Sci. Rep..

[25] Feng Y., Chen C., Han Y., Chen Z., Lu X., Liang F., Li S., Qin W., Lin S. (2016). Expanding CRISPR/Cas9 Genome Editing Capacity in Zebrafish Using SaCas9. G3.

[26] Zhang X., Liang P., Ding C., Zhang Z., Zhou J., Xie X., Huang R., Sun Y., Sun H., Zhang J. (2016). Efficient Production of Gene-Modified Mice using Staphylococcus aureus Cas9. Sci. Rep..

[27] Nishimasu H., Cong L., Yan W.X., Ran F.A., Zetsche B., Li Y., Kurabayashi A., Ishitani R., Zhang F., Nureki O. (2015). Crystal Structure of Staphylococcus aureus Cas9. Cell.

[28] Zetsche B., Gootenberg J.S., Abudayyeh O.O., Slaymaker I.M., Makarova K.S., Essletzbichler P., Volz S.E., Joung J., van der Oost J., Regev A. (2015). Cpf1 is a single RNA-guided endonuclease of a class 2 CRISPR-Cas system. Cell.

[29] Kim D., Kim J., Hur J.K., Been K.W., Yoon S.H., Kim J.S. (2016). Genome-wide analysis reveals specificities of Cpf1 endonucleases in human cells. Nat. Biotechnol..

[30] Yamano T., Nishimasu H., Zetsche B., Hirano H., Slaymaker I.M., Li Y., Fedorova I., Nakane T., Makarova K.S., Koonin E.V. (2016). Crystal Structure of Cpf1 in Complex with Guide RNA and Target DNA. Cell.

[31] Zetsche B., Heidenreich M., Mohanraju P., Fedorova I., Kneppers J., DeGennaro E.M., Winblad N., Choudhury S.R., Abudayyeh O.O., Gootenberg J.S. (2017). Multiplex gene editing by CRISPR-Cpf1 using a single crRNA array. Nat. Biotechnol..

[32] Endo A., Masafumi M., Kaya H., Toki S. (2016). Efficient targeted mutagenesis of rice and tobacco genomes using Cpf1 from *Francisella novicida*. Sci. Rep..

[33] Tang X., Lowder L.G., Zhang T., Malzahn A.A., Zheng X., Voytas D.F., Zhong Z., Chen Y., Ren Q., Li Q. (2017). A CRISPR-Cpf1 system for efficient genome editing and transcriptional repression in plants. Nat. Plants.

[34] Ungerer J., Pakrasi H.B. (2016). Cpf1 Is A Versatile Tool for CRISPR Genome Editing across Diverse Species of Cyanobacteria. Sci. Rep..

[35] Kim Y., Cheong S.A., Lee J.G., Lee S.W., Lee M.S., Baek I.J., Sung Y.H. (2016). Generation of knockout mice by Cpf1-mediated gene targeting. Nat. Biotechnol..

[36] Fonfara I., Richter H., Bratovic M., Le Rhun A., Charpentier E. (2016). The CRISPR-associated DNA-cleaving enzyme Cpf1 also processes precursor CRISPR RNA. Nature.

[37] Sampson T.R., Saroj S.D., Llewellyn A.C., Tzeng Y.L., Weiss D.S. (2013). A CRISPR/Cas system mediates bacterial innate immune evasion and virulence. Nature.

[38] Price A.A., Sampson T.R., Ratner H.K., Grakoui A., Weiss D.S. (2015). Cas9-mediated targeting of viral RNA in eukaryotic cells. Proc. Natl. Acad. Sci. USA.

[39] Shmakov S., Abudayyeh O.O., Makarova K.S., Wolf Y.I., Gootenberg J.S., Semenova E., Minakhin L., Joung J., Konermann S., Severinov K. (2015). Discovery and Functional Characterization of Diverse Class 2 CRISPR-Cas Systems. Mol. Cell.

[40] Abudayyeh O.O., Gootenberg J.S., Konermann S., Joung J., Slaymaker I.M., Cox D.B., Shmakov S., Makarova K.S., Semenova E., Minakhin L. (2016). C2c2 is a single-component programmable RNA-guided RNA-targeting CRISPR effector. Science.

[41] East-Seletsky A., O’Connell M.R., Knight S.C., Burstein D., Cate J.H., Tjian R., Doudna J.A. (2016). Two distinct RNase activities of CRISPR-C2c2 enable guide-RNA processing and RNA detection. Nature.

[42] Liu L., Li X., Wang J., Wang M., Chen P., Yin M., Li J., Sheng G., Wang Y. (2017). Two Distant Catalytic Sites Are Responsible for C2c2 RNase Activities. Cell.

[43] Liu L., Chen P., Wang M., Li X., Wang J., Yin M., Wang Y. (2017). C2c1-sgRNA Complex Structure Reveals RNA-Guided DNA Cleavage Mechanism. Mol. Cell.

[44] Yang H., Gao P., Rajashankar K.R., Patel D.J. (2016). PAM-Dependent Target DNA Recognition and Cleavage by C2c1 CRISPR-Cas Endonuclease. Cell.

[45] Abudayyeh O.O., Gootenberg J.S., Essletzbichler P., Han S., Joung J., Belanto J.J., Verdine V., Cox D.B.T., Kellner M.J., Regev A. (2017). RNA targeting with CRISPR-Cas13. Nature.

[46] Cox D.B.T., Gootenberg J.S., Abudayyeh O.O., Franklin B., Kellner M.J., Joung J., Zhang F. (2017). RNA editing with CRISPR-Cas13. Science.

[47] Chan D.C., Kim P.S. (1998). HIV entry and its inhibition. Cell.

[48] Zheng Y.H., Lovsin N., Peterlin B.M. (2005). Newly identified host factors modulate HIV replication. Immunol. Lett..

[49] Finzi D., Blankson J., Siliciano J.D., Margolick J.B., Chadwick K., Pierson T., Smith K., Lisziewicz J., Lori F., Flexner C. (1999). Latent infection of CD4+ T cells provides a mechanism for lifelong persistence of HIV-1, even in patients on effective combination therapy. Nat. Med..

[50] Kim S.Y., Byrn R., Groopman J., Baltimore D. (1989). Temporal aspects of DNA and RNA synthesis during human immunodeficiency virus infection: Evidence for differential gene expression. J. Virol..

[51] Pomerantz R.J., Trono D., Feinberg M.B., Baltimore D. (1990). Cells nonproductively infected with HIV-1 exhibit an aberrant pattern of viral RNA expression: A molecular model for latency. Cell.

[52] (1982). Update on acquired immune deficiency syndrome (AIDS)—United States. Morb. Mortal. Wkly. Rep..

[53] Bayer R., Levine C., Murray T.H. (1983). Guidelines for Confidentiality in Research on AIDS. AIDS Res..

[54] Palella F.J., Delaney K.M., Moorman A.C., Loveless M.O., Fuhrer J., Satten G.A., Aschman D.J., Holmberg S.D. (1998). Declining morbidity and mortality among patients with advanced human immunodeficiency virus infection. HIV Outpatient Study Investigators. N. Engl. J. Med..

[55] Durand C.M., Blankson J.N., Siliciano R.F. (2012). Developing strategies for HIV-1 eradication. Trends Immunol..

[56] Siliciano J.D., Siliciano R.F. (2013). HIV-1 eradication strategies: Design and assessment. Curr. Opin. HIV AIDS.

[57] Blas-Garcia A., Apostolova N., Esplugues J.V. (2011). Oxidative stress and mitochondrial impairment after treatment with anti-HIV drugs: Clinical implications. Curr. Pharm. Des..

[58] Allers K., Hutter G., Hofmann J., Loddenkemper C., Rieger K., Thiel E., Schneider T. (2011). Evidence for the cure of HIV infection by CCR5 Delta 32/Delta 32 stem cell transplantation. Blood.

[59] Hutter G., Nowak D., Mossner M., Ganepola S., Muessig A., Allers K., Schneider T., Hofmann J., Kuecherer C., Blau O. (2009). Long-Term Control of HIV by CCR5 Delta32/Delta32 Stem-Cell Transplantaion. N. Engl. J. Med..

[60] Ye L., Wang J., Beyer A.I., Teque F., Cradick T.J., Qi Z., Chang J.C., Bao G., Muench M.O., Yu J. (2014). Seamless modification of wild-type induced pluripotent stem cells to the natural CCR5Delta32 mutation confers resistance to HIV infection. Proc. Natl. Acad. Sci. USA.

[61] Wang W., Ye C., Liu J., Zhang D., Kimata J.T., Zhou P. (2014). CCR5 gene disruption via lentiviral vectors expressing Cas9 and single guided RNA renders cells resistant to HIV-1 infection. PLoS ONE.

[62] Li C., Guan X., Du T., Jin W., Wu B., Liu Y., Wang P., Hu B., Griffin G.E., Shattock R.J. (2015). Inhibition of HIV-1 infection of primary CD4+ T-cells by gene editing of CCR5 using adenovirus-delivered CRISPR/Cas9. J. Gener. Virol..

[63] Mandal P.K., Ferreira L.M., Collins R., Meissner T.B., Boutwell C.L., Friesen M., Vrbanac V., Garrison B.S., Stortchevoi A., Bryder D. (2014). Efficient ablation of genes in human hematopoietic stem and effector cells using CRISPR/Cas9. Cell Stem Cell.

[64] Xu L., Yang H., Gao Y., Chen Z., Xie L., Liu Y., Liu Y., Wang X., Li H., Lai W. (2017). CRISPR/Cas9-Mediated CCR5 Ablation in Human Hematopoietic Stem/Progenitor Cells Confers HIV-1 Resistance In Vivo. Mol. Ther. J. Am. Soc. Gene Ther..

[65] Feng Y., Broder C.C., Kennedy P.E., Berger E.A. (1996). HIV-1 entry cofactor: Functional cDNA cloning of a seven-transmembrane, G protein-coupled receptor. Science.

[66] Deng H.K., Liu R., Ellmeier W., Choe S., Unutmaz D., Burkhart M., DiMarzio P., Marmon S., Sutton R.E., Hill C.M. (1996). Identification of a major co-receptor for primary isolates of HIV-1. Nature.

[67] Connor R.I., Sheridan K.E., Ceradini D., Choe S., Landau N.R. (1997). Change in coreceptor use correlates with disease progression in HIV-1-infected individuals. J. Exp. Med..

[68] Huang W., Toma J., Stawiski E., Fransen S., Wrin T., Parkin N., Whitcomb J.M., Coakley E., Hecht F.M., Deeks S.G. (2009). Characterization of human immunodeficiency virus type 1 populations containing CXCR4-using variants from recently infected individuals. AIDS Res. Hum. Retroviruses.

[69] Hou P., Chen S., Wang S., Yu X., Chen Y., Jiang M., Zhuang K., Ho W., Hou W., Huang J. (2015). Genome editing of CXCR4 by CRISPR/cas9 confers cells resistant to HIV-1 infection. Sci. Rep..

[70] Hultquist J.F., Schumann K., Woo J.M., Manganaro L., McGregor M.J., Doudna J., Simon V., Krogan N.J., Marson A. (2016). A Cas9 Ribonucleoprotein Platform for Functional Genetic Studies of HIV-Host Interactions in Primary Human T Cells. Cell Rep..

[71] Yu S., Yao Y., Xiao H., Li J., Liu Q., Yang Y., Adah D., Lu J., Zhao S., Qin L. (2017). Simultaneous knockout of *CXCR4* and *CCR5* genes in CD4+ T cells via CRISPR/Cas9 confers resistance to both X4- and R5-tropic HIV-1 infection. Hum. Gene Ther..

[72] Liu Z., Chen S., Jin X., Wang Q., Yang K., Li C., Xiao Q., Hou P., Liu S., Wu S. (2017). Genome editing of the HIV co-receptors CCR5 and CXCR4 by CRISPR-Cas9 protects CD4+ T cells from HIV-1 infection. Cell Biosci..

[73] Ebina H., Misawa N., Kanemura Y., Koyanagi Y. (2013). Harnessing the CRISPR/Cas9 system to disrupt latent HIV-1 provirus. Sci. Rep..

[74] Hu W., Kaminski R., Yang F., Zhang Y., Cosentino L., Li F., Luo B., Alvarez-Carbonell D., Garcia-Mesa Y., Karn J. (2014). RNA-directed gene editing specifically eradicates latent and prevents new HIV-1 infection. Proc. Natl. Acad. Sci. USA.

[75] Zhu W., Lei R., Le Duff Y., Li J., Guo F., Wainberg M.A., Liang C. (2015). The CRISPR/Cas9 system inactivates latent HIV-1 proviral DNA. Retrovirology.

[76] Liao H.K., Gu Y., Diaz A., Marlett J., Takahashi Y., Li M., Suzuki K., Xu R., Hishida T., Chang C.J. (2015). Use of the CRISPR/Cas9 system as an intracellular defense against HIV-1 infection in human cells. Nat. Commun..

[77] Maertens G., Cherepanov P., Pluymers W., Busschots K., de Clercq E., Debyser Z., Engelborghs Y. (2003). LEDGF/p75 is essential for nuclear and chromosomal targeting of HIV-1 integrase in human cells. J. Biol. Chem..

[78] Christ F., Debyser Z. (2013). The LEDGF/p75 integrase interaction, a novel target for anti-HIV therapy. Virology.

[79] Fadel H.J., Morrison J.H., Saenz D.T., Fuchs J.R., Kvaratskhelia M., Ekker S.C., Poeschla E.M. (2014). TALEN knockout of the PSIP1 gene in human cells: Analyses of HIV-1 replication and allosteric integrase inhibitor mechanism. J. Virol..

[80] Brass A.L., Dykxhoorn D.M., Benita Y., Yan N., Engelman A., Xavier R.J., Lieberman J., Elledge S.J. (2008). Identification of host proteins required for HIV infection through a functional genomic screen. Science.

[81] Schaller T., Ocwieja K.E., Rasaiyaah J., Price A.J., Brady T.L., Roth S.L., Hue S., Fletcher A.J., Lee K., KewalRamani V.N. (2011). HIV-1 capsid-cyclophilin interactions determine nuclear import pathway, integration targeting and replication efficiency. PLoS Pathog..

[82] Price A.J., Fletcher A.J., Schaller T., Elliott T., Lee K., KewalRamani V.N., Chin J.W., Towers G.J., James L.C. (2012). CPSF6 defines a conserved capsid interface that modulates HIV-1 replication. PLoS Pathog..

[83] Park R.J., Wang T., Koundakjian D., Hultquist J.F., Lamothe-Molina P., Monel B., Schumann K., Yu H., Krupzcak K.M., Garcia-Beltran W. (2017). A genome-wide CRISPR screen identifies a restricted set of HIV host dependency factors. Nat. Genet..

[84] Konig R., Zhou Y., Elleder D., Diamond T.L., Bonamy G.M., Irelan J.T., Chiang C.Y., Tu B.P., De Jesus P.D., Lilley C.E. (2008). Global analysis of host-pathogen interactions that regulate early-stage HIV-1 replication. Cell.

[85] Zhou H., Xu M., Huang Q., Gates A.T., Zhang X.D., Castle J.C., Stec E., Ferrer M., Strulovici B., Hazuda D.J. (2008). Genome-scale RNAi screen for host factors required for HIV replication. Cell Host Microbe.

[86] Gori J.L., Hsu P.D., Maeder M.L., Shen S., Welstead G.G., Bumcrot D. (2015). Delivery and Specificity of CRISPR-Cas9 Genome Editing Technologies for Human Gene Therapy. Hum. Gene Ther..

[87] Choi J.G., Dang Y., Abraham S., Ma H., Zhang J., Guo H., Cai Y., Mikkelsen J.G., Wu H., Shankar P. (2016). Lentivirus pre-packed with Cas9 protein for safer gene editing. Gene Ther..

[88] Sather B.D., Romano Ibarra G.S., Sommer K., Curinga G., Hale M., Khan I.F., Singh S., Song Y., Gwiazda K., Sahni J. (2015). Efficient modification of CCR5 in primary human hematopoietic cells using a megaTAL nuclease and AAV donor template. Sci. Transl. Med..

[89] Wang J., DeClercq J.J., Hayward S.B., Li P.W., Shivak D.A., Gregory P.D., Lee G., Holmes M.C. (2016). Highly efficient homology-driven genome editing in human T cells by combining zinc-finger nuclease mRNA and AAV6 donor delivery. Nucleic Acids Res..

[90] Wang J., Exline C.M., DeClercq J.J., Llewellyn G.N., Hayward S.B., Li P.W., Shivak D.A., Surosky R.T., Gregory P.D., Holmes M.C. (2015). Homology-driven genome editing in hematopoietic stem and progenitor cells using ZFN mRNA and AAV6 donors. Nat. Biotechnol..

[91] Wang Q., Chen S., Xiao Q., Liu Z., Liu S., Hou P., Zhou L., Hou W., Ho W., Li C. (2017). Genome modification of CXCR4 by Staphylococcus aureus Cas9 renders cells resistance to HIV-1 infection. Retrovirology.

[92] Yin C., Zhang T., Qu X., Zhang Y., Putatunda R., Xiao X., Li F., Xiao W., Zhao H., Dai S. (2017). In Vivo Excision of HIV-1 Provirus by saCas9 and Multiplex Single-Guide RNAs in Animal Models. Mol. Ther..

[93] Deeks S.G., Autran B., Berkhout B., Benkirane M., Cairns S., Chomont N., Chun T.W., Churchill M., Di Mascio M., Katlama C. (2012). Towards an HIV cure: A global scientific strategy. Nat. Rev. Immunol..

[94] Richman D.D., Margolis D.M., Delaney M., Greene W.C., Hazuda D., Pomerantz R.J. (2009). The challenge of finding a cure for HIV infection. Science.

[95] Deeks S.G. (2012). HIV: Shock and kill. Nature.

[96] Archin N.M., Liberty A.L., Kashuba A.D., Choudhary S.K., Kuruc J.D., Crooks A.M., Parker D.C., Anderson E.M., Kearney M.F., Strain M.C. (2012). Administration of vorinostat disrupts HIV-1 latency in patients on antiretroviral therapy. Nature.

[97] Zhang Y., Yin C., Zhang T., Li F., Yang W., Kaminski R., Fagan P.R., Putatunda R., Young W.B., Khalili K. (2015). CRISPR/gRNA-directed synergistic activation mediator (SAM) induces specific, persistent and robust reactivation of the HIV-1 latent reservoirs. Sci. Rep..

[98] Saayman S.M., Lazar D.C., Scott T.A., Hart J.R., Takahashi M., Burnett J.C., Planelles V., Morris K.V., Weinberg M.S. (2016). Potent and Targeted Activation of Latent HIV-1 Using the CRISPR/dCas9 Activator Complex. Mol. Ther. J. Am. Soc. Gene Ther..

[99] Limsirichai P., Gaj T., Schaffer D.V. (2016). CRISPR-mediated Activation of Latent HIV-1 Expression. Mol. Ther. J. Am. Soc. Gene Ther..

[100] Ji H., Jiang Z., Lu P., Ma L., Li C., Pan H., Fu Z., Qu X., Wang P., Deng J. (2016). Specific Reactivation of Latent HIV-1 by dCas9-SunTag-VP64-mediated Guide RNA Targeting the HIV-1 Promoter. Mol. Ther. J. Am. Soc. Gene Ther..

[101] Stremlau M., Owens C.M., Perron M.J., Kiessling M., Autissier P., Sodroski J. (2004). The cytoplasmic body component TRIM5alpha restricts HIV-1 infection in Old World monkeys. Nature.

[102] Li Y., Li X., Stremlau M., Lee M., Sodroski J. (2006). Removal of arginine 332 allows human TRIM5alpha to bind human immunodeficiency virus capsids and to restrict infection. J. Virol..

[103] Anderson J., Akkina R. (2008). Human immunodeficiency virus type 1 restriction by human-rhesus chimeric tripartite motif 5alpha (TRIM 5alpha) in CD34(+) cell-derived macrophages in vitro and in T cells in vivo in severe combined immunodeficient (SCID-hu) mice transplanted with human fetal tissue. Hum. Gene Ther..

[104] Pham Q.T., Bouchard A., Grutter M.G., Berthoux L. (2010). Generation of human TRIM5alpha mutants with high HIV-1 restriction activity. Gene Ther..

[105] Neagu M.R., Ziegler P., Pertel T., Strambio-de-Castillia C., Grutter C., Martinetti G., Mazzucchelli L., Grutter M., Manz M.G., Luban J. (2009). Potent inhibition of HIV-1 by TRIM5-cyclophilin fusion proteins engineered from human components. J. Clin. Investig..

[106] Sheehy A.M., Gaddis N.C., Choi J.D., Malim M.H. (2002). Isolation of a human gene that inhibits HIV-1 infection and is suppressed by the viral Vif protein. Nature.

[107] Xu H., Svarovskaia E.S., Barr R., Zhang Y., Khan M.A., Strebel K., Pathak V.K. (2004). A single amino acid substitution in human APOBEC3G antiretroviral enzyme confers resistance to HIV-1 virion infectivity factor-induced depletion. Proc. Natl. Acad. Sci. USA.

[108] Schrofelbauer B., Chen D., Landau N.R. (2004). A single amino acid of APOBEC3G controls its species-specific interaction with virion infectivity factor (Vif). Proc. Natl. Acad. Sci. USA.

[109] Neil S.J., Zang T., Bieniasz P.D. (2008). Tetherin inhibits retrovirus release and is antagonized by HIV-1 Vpu. Nature.

[110] Van Damme N., Goff D., Katsura C., Jorgenson R.L., Mitchell R., Johnson M.C., Stephens E.B., Guatelli J. (2008). The interferon-induced protein BST-2 restricts HIV-1 release and is downregulated from the cell surface by the viral Vpu protein. Cell Host Microbe.

[111] Gupta R.K., Hue S., Schaller T., Verschoor E., Pillay D., Towers G.J. (2009). Mutation of a single residue renders human tetherin resistant to HIV-1 Vpu-mediated depletion. PLoS Pathog..

[112] Hrecka K., Hao C., Gierszewska M., Swanson S.K., Kesik-Brodacka M., Srivastava S., Florens L., Washburn M.P., Skowronski J. (2011). Vpx relieves inhibition of HIV-1 infection of macrophages mediated by the SAMHD1 protein. Nature.

[113] Laguette N., Sobhian B., Casartelli N., Ringeard M., Chable-Bessia C., Segeral E., Yatim A., Emiliani S., Schwartz O., Benkirane M. (2011). SAMHD1 is the dendritic- and myeloid-cell-specific HIV-1 restriction factor counteracted by Vpx. Nature.

[114] Descours B., Cribier A., Chable-Bessia C., Ayinde D., Rice G., Crow Y., Yatim A., Schwartz O., Laguette N., Benkirane M. (2012). SAMHD1 restricts HIV-1 reverse transcription in quiescent CD4(+) T-cells. Retrovirology.

[115] Lahouassa H., Daddacha W., Hofmann H., Ayinde D., Logue E.C., Dragin L., Bloch N., Maudet C., Bertrand M., Gramberg T. (2012). SAMHD1 restricts the replication of human immunodeficiency virus type 1 by depleting the intracellular pool of deoxynucleoside triphosphates. Nat. Immunol..

[116] Wu L. (2012). SAMHD1: A new contributor to HIV-1 restriction in resting CD4+ T-cells. Retrovirology.

[117] Baldauf H.M., Pan X., Erikson E., Schmidt S., Daddacha W., Burggraf M., Schenkova K., Ambiel I., Wabnitz G., Gramberg T. (2012). SAMHD1 restricts HIV-1 infection in resting CD4(+) T cells. Nat. Med..

[118] Kane M., Yadav S.S., Bitzegeio J., Kutluay S.B., Zang T., Wilson S.J., Schoggins J.W., Rice C.M., Yamashita M., Hatziioannou T. (2013). MX2 is an interferon-induced inhibitor of HIV-1 infection. Nature.

[119] Goujon C., Moncorge O., Bauby H., Doyle T., Ward C.C., Schaller T., Hue S., Barclay W.S., Schulz R., Malim M.H. (2013). Human MX2 is an interferon-induced post-entry inhibitor of HIV-1 infection. Nature.

[120] Voit R.A., McMahon M.A., Sawyer S.L., Porteus M.H. (2013). Generation of an HIV resistant T-cell line by targeted “stacking” of restriction factors. Mol. Ther. J. Am. Soc. Gene Ther..

[121] Bogerd H.P., Kornepati A.V., Marshall J.B., Kennedy E.M., Cullen B.R. (2015). Specific induction of endogenous viral restriction factors using CRISPR/Cas-derived transcriptional activators. Proc. Natl. Acad. Sci. USA.

[122] Usami Y., Wu Y., Gottlinger H.G. (2015). SERINC3 and SERINC5 restrict HIV-1 infectivity and are counteracted by Nef. Nature.

[123] Rosa A., Chande A., Ziglio S., de Sanctis V., Bertorelli R., Goh S.L., McCauley S.M., Nowosielska A., Antonarakis S.E., Luban J. (2015). HIV-1 Nef promotes infection by excluding SERINC5 from virion incorporation. Nature.

[124] Lin G., Zhang K., Li J. (2015). Application of CRISPR/Cas9 Technology to HBV. Int. J. Mol. Sci..

[125] Peng C., Lu M., Yang D. (2015). CRISPR/Cas9-based tools for targeted genome editing and replication control of HBV. Virol. Sin..

[126] Dong C., Qu L., Wang H., Wei L., Dong Y., Xiong S. (2015). Targeting hepatitis B virus cccDNA by CRISPR/Cas9 nuclease efficiently inhibits viral replication. Antivir. Res..

[127] Jiang C., Mei M., Li B., Zhu X., Zu W., Tian Y., Wang Q., Guo Y., Dong Y., Tan X. (2017). A non-viral CRISPR/Cas9 delivery system for therapeutically targeting HBV DNA and pcsk9 in vivo. Cell Res..

[128] Karimova M., Beschorner N., Dammermann W., Chemnitz J., Indenbirken D., Bockmann J.H., Grundhoff A., Luth S., Buchholz F., Schulze zur Wiesch J. (2015). CRISPR/Cas9 nickase-mediated disruption of hepatitis B virus open reading frame S and X. Sci. Rep..

[129] Kennedy E.M., Bassit L.C., Mueller H., Kornepati A.V., Bogerd H.P., Nie T., Chatterjee P., Javanbakht H., Schinazi R.F., Cullen B.R. (2015). Suppression of hepatitis B virus DNA accumulation in chronically infected cells using a bacterial CRISPR/Cas RNA-guided DNA endonuclease. Virology.

[130] Li H., Sheng C., Wang S., Yang L., Liang Y., Huang Y., Liu H., Li P., Yang C., Yang X. (2017). Removal of Integrated Hepatitis B Virus DNA Using CRISPR-Cas9. Front. Cell. Infect. Microbiol..

[131] Lin S.R., Yang H.C., Kuo Y.T., Liu C.J., Yang T.Y., Sung K.C., Lin Y.Y., Wang H.Y., Wang C.C., Shen Y.C. (2014). The CRISPR/Cas9 System Facilitates Clearance of the Intrahepatic HBV Templates In Vivo. Mol. Ther. Nucleic Acids.

[132] Liu X., Hao R., Chen S., Guo D., Chen Y. (2015). Inhibition of hepatitis B virus by the CRISPR/Cas9 system via targeting the conserved regions of the viral genome. J. Gener. Virol..

[133] Pankowicz F.P., Jarrett K.E., Lagor W.R., Bissig K.D. (2017). CRISPR/Cas9: At the cutting edge of hepatology. Gut.

[134] Ramanan V., Shlomai A., Cox D.B., Schwartz R.E., Michailidis E., Bhatta A., Scott D.A., Zhang F., Rice C.M., Bhatia S.N. (2015). CRISPR/Cas9 cleavage of viral DNA efficiently suppresses hepatitis B virus. Sci. Rep..

[135] Seeger C., Sohn J.A. (2014). Targeting Hepatitis B Virus With CRISPR/Cas9. Mol. Ther. Nucleic Acids.

[136] Seeger C., Sohn J.A. (2016). Complete Spectrum of CRISPR/Cas9-induced Mutations on HBV cccDNA. Mol. Ther. J. Am. Soc. Gene Ther..

[137] Wang J., Chen R., Zhang R., Ding S., Zhang T., Yuan Q., Guan G., Chen X., Zhang T., Zhuang H. (2017). The gRNA-miRNA-gRNA Ternary Cassette Combining CRISPR/Cas9 with RNAi Approach Strongly Inhibits Hepatitis B Virus Replication. Theranostics.

[138] Wang J., Xu Z.W., Liu S., Zhang R.Y., Ding S.L., Xie X.M., Long L., Chen X.M., Zhuang H., Lu F.M. (2015). Dual gRNAs guided CRISPR/Cas9 system inhibits hepatitis B virus replication. World J. Gastroenterol..

[139] Zhen S., Hua L., Liu Y.H., Gao L.C., Fu J., Wan D.Y., Dong L.H., Song H.F., Gao X. (2015). Harnessing the clustered regularly interspaced short palindromic repeat (CRISPR)/CRISPR-associated Cas9 system to disrupt the hepatitis B virus. Gene Ther..

[140] Zhu W., Xie K., Xu Y., Wang L., Chen K., Zhang L., Fang J. (2016). CRISPR/Cas9 produces anti-hepatitis B virus effect in hepatoma cells and transgenic mouse. Virus Res..

[141] Yan H., Zhong G., Xu G., He W., Jing Z., Gao Z., Huang Y., Qi Y., Peng B., Wang H. (2012). Sodium taurocholate cotransporting polypeptide is a functional receptor for human hepatitis B and D virus. eLife.

[142] Watashi K., Sluder A., Daito T., Matsunaga S., Ryo A., Nagamori S., Iwamoto M., Nakajima S., Tsukuda S., Borroto-Esoda K. (2014). Cyclosporin A and its analogs inhibit hepatitis B virus entry into cultured hepatocytes through targeting a membrane transporter NTCP. Hepatology.

[143] Nkongolo S., Ni Y., Lempp F.A., Kaufman C., Lindner T., Essernobis K., Lohmann V., Mier W., Mehrle S., Urban S. (2014). Cyclosporin A inhibits hepatitis B and hepatitis D virus entry by cyclophilin-independent interference with the NTCP receptor. J. Hepatol..

[144] Gripon P., Cannie I., Urban S. (2005). Efficient inhibition of hepatitis B virus infection by acylated peptides derived from the large viral surface protein. J. Virol..

[145] Petersen J., Dandri M., Mier W., Lãtgehetmann M., Volz T., von Weizsäcker F., Haberkorn U., Fischer L., Pollok J.M., Erbes B. (2008). Prevention of hepatitis B virus infection in vivo by entry inhibitors derived from the large envelope protein. Nat. Biotechnol..

[146] Volz T., Allweiss L., Ben M.M., Warlich M., Lohse A.W., Pollok J.M., Alexandrov A., Urban S., Petersen J., Lütgehetmann M. (2013). The entry inhibitor Myrcludex-B efficiently blocks intrahepatic virus spreading in humanized mice previously infected with hepatitis B virus. J. Hepatol..

[147] Hoh A., Heeg M., Ni Y., Schuch A., Binder B., Hennecke N., Blum H.E., Nassal M., Protzer U., Hofmann M. (2015). Hepatitis B Virus-Infected HepG2hNTCP Cells Serve as a Novel Immunological Tool To Analyze the Antiviral Efficacy of CD8+ T Cells In Vitro. J. Virol..

[148] Stahl M., Retzlaff M., Nassal M., Beck J. (2007). Chaperone activation of the hepadnaviral reverse transcriptase for template RNA binding is established by the Hsp70 and stimulated by the Hsp90 system. Nucleic Acids Res..

[149] Wang Y.P., Liu F., He H.W., Han Y.X., Peng Z.G., Li B.W., You X.F., Song D.Q., Li Z.R., Yu L.Y. (2010). Heat Stress Cognate 70 Host Protein as a Potential Drug Target against Drug Resistance in Hepatitis B Virus. Antimicrob. Agents Chemother..

[150] Zhou Y.B., Wang Y.F., Zhang Y., Zheng L.Y., Yang X.A., Wang N., Jiang J.H., Ma F., Yin D.T., Sun C.Y. (2012). In vitro activity of cepharanthine hydrochloride against clinical wild-type and lamivudine-resistant hepatitis B virus isolates. Eur. J. Pharmacol..

[151] Shim H.Y., Quan X., Yi Y.S., Jung G. (2011). Heat shock protein 90 facilitates formation of the HBV capsid via interacting with the HBV core protein dimers. Virology.

[152] Hu J., Seeger C. (1996). Hsp90 is Required for the Activity of a Hepatitis B Virus Reverse Transcriptase. Proc. Natl. Acad. Sci. USA.

[153] Li W., Miao X., Qi Z., Zeng W., Liang J., Liang Z. (2010). Hepatitis B virus X protein upregulates HSP90alpha expression via activation of c-Myc in human hepatocarcinoma cell line, HepG2. Virol. J..

[154] Königer C., Wingert I., Marsmann M., Rösler C., Beck J., Nassal M. (2014). Involvement of the host DNA-repair enzyme TDP2 in formation of the covalently closed circular DNA persistence reservoir of hepatitis B viruses. Proc. Natl. Acad. Sci. USA.

[155] Cui X., Mcallister R., Boregowda R., Ji A.S., Ledesma F.C., Caldecott K.W., Seeger C., Hu J. (2015). Does Tyrosyl DNA Phosphodiesterase-2 Play a Role in Hepatitis B Virus Genome Repair?. PLoS ONE.

[156] Hartmann-Stuhler C., Prange R. (2001). Hepatitis B virus large envelope protein interacts with gamma2-adaptin, a clathrin adaptor-related protein. J. Virol..

[157] Rost M., Mann S., Lambert C., Döring T., Thomé N., Prange R. (2006). Gamma-adaptin, a novel ubiquitin-interacting adaptor, and Nedd4 ubiquitin ligase control hepatitis B virus maturation. J. Biol. Chem..

[158] Lv M., Zhang B., Shi Y., Han Z., Zhang Y., Zhou Y., Zhang W., Niu J., Yu X.F. (2015). Identification of BST-2/tetherin-induced hepatitis B virus restriction and hepatocyte-specific BST-2 inactivation. Sci. Rep..

[159] Yan R., Zhao X., Cai D., Liu Y., Block T.M., Guo J.T., Guo H. (2015). The Interferon-Inducible Protein Tetherin Inhibits Hepatitis B Virus Virion Secretion. J. Virol..

[160] Miyakawa K., Matsunaga S., Watashi K., Sugiyama M., Kimura H., Yamamoto N., Mizokami M., Wakita T., Ryo A. (2015). Molecular dissection of HBV evasion from restriction factor tetherin: A new perspective for antiviral cell therapy. Oncotarget.

[161] Fehrmann F., Laimins L.A. (2003). Human papillomaviruses: Targeting differentiating epithelial cells for malignant transformation. Oncogene.

[162] Ghosh I., Mittal S., Banerjee D., Singh P., Dasgupta S., Chatterjee S., Biswas J., Panda C., Basu P. (2014). Study of accuracy of colposcopy in VIA and HPV detection-based cervical cancer screening program. Aust. N. Z. J. Obstet. Gynaecol..

[163] Munoz N., Kjaer S.K., Sigurdsson K., Iversen O.E., Hernandez-Avila M., Wheeler C.M., Perez G., Brown D.R., Koutsky L.A., Tay E.H. (2010). Impact of human papillomavirus (HPV)-6/11/16/18 vaccine on all HPV-associated genital diseases in young women. J. Natl. Cancer Inst..

[164] Moody C.A., Laimins L.A. (2010). Human papillomavirus oncoproteins: Pathways to transformation. Nat. Rev. Cancer.

[165] Zur Hausen H. (2002). Papillomaviruses and cancer: From basic studies to clinical application. Nat. Rev. Cancer.

[166] Kennedy E.M., Kornepati A.V., Goldstein M., Bogerd H.P., Poling B.C., Whisnant A.W., Kastan M.B., Cullen B.R. (2014). Inactivation of the human papillomavirus E6 or E7 gene in cervical carcinoma cells by using a bacterial CRISPR/Cas RNA-guided endonuclease. J. Virol..

[167] Zhen S., Hua L., Takahashi Y., Narita S., Liu Y.H., Li Y. (2014). In vitro and in vivo growth suppression of human papillomavirus 16-positive cervical cancer cells by CRISPR/Cas9. Biochem. Biophys. Res. Commun..

[168] Porter S.S., Stepp W.H., Stamos J.D., McBride A.A. (2017). Host cell restriction factors that limit transcription and replication of human papillomavirus. Virus Res..

[169] Das D., Smith N., Wang X., Morgan I.M. (2017). The Deacetylase SIRT1 Regulates the Replication Properties of Human Papillomavirus 16 E1 and E2. J. Virol..

[170] McKinney C.C., Kim M.J., Chen D., McBride A.A. (2016). Brd4 Activates Early Viral Transcription upon Human Papillomavirus 18 Infection of Primary Keratinocytes. mBio.

[171] Meuris F., Carthagena L., Jaracz-Ros A., Gaudin F., Cutolo P., Deback C., Xue Y., Thierry F., Doorbar J., Bachelerie F. (2016). The CXCL12/CXCR4 Signaling Pathway: A New Susceptibility Factor in Human Papillomavirus Pathogenesis. PLoS Pathog..

[172] Zhang W., Hong S., Maniar K.P., Cheng S., Jie C., Rademaker A.W., Krensky A.M., Clayberger C. (2016). KLF13 regulates the differentiation-dependent human papillomavirus life cycle in keratinocytes through STAT5 and IL-8. Oncogene.

[173] Gunasekharan V.K., Li Y., Andrade J., Laimins L.A. (2016). Post-Transcriptional Regulation of KLF4 by High-Risk Human Papillomaviruses Is Necessary for the Differentiation-Dependent Viral Life Cycle. PLoS Pathog..

[174] DeSmet M., Kanginakudru S., Rietz A., Wu W.H., Roden R., Androphy E.J. (2016). The Replicative Consequences of Papillomavirus E2 Protein Binding to the Origin Replication Factor ORC2. PLoS Pathog..

[175] Peta E., Cappellesso R., Masi G., Sinigaglia A., Trevisan M., Grassi A., Di Camillo B., Vassarotto E., Fassina A., Palu G. (2017). Down-regulation of microRNA-146a is associated with high-risk human papillomavirus infection and epidermal growth factor receptor overexpression in penile squamous cell carcinoma. Hum. Pathol..

[176] Tzellos S., Farrell P.J. (2012). Epstein-barr virus sequence variation-biology and disease. Pathogens.

[177] Raab-Traub N. (2012). Novel mechanisms of EBV-induced oncogenesis. Curr. Opin. Virol..

[178] Taylor G.S., Long H.M., Brooks J.M., Rickinson A.B., Hislop A.D. (2015). The immunology of Epstein-Barr virus-induced disease. Ann. Rev. Immunol..

[179] Ok C.Y., Li L., Young K.H. (2015). EBV-driven B-cell lymphoproliferative disorders: From biology, classification and differential diagnosis to clinical management. Exp. Mol. Med..

[180] Price A.M., Luftig M.A. (2014). Dynamic Epstein-Barr virus gene expression on the path to B-cell transformation. Adv. Virus Res..

[181] Wang J., Quake S.R. (2014). RNA-guided endonuclease provides a therapeutic strategy to cure latent herpesviridae infection. Proc. Natl. Acad. Sci. USA.

[182] Yuen K.S., Chan C.P., Wong N.H., Ho C.H., Ho T.H., Lei T., Deng W., Tsao S.W., Chen H., Kok K.H. (2015). CRISPR/Cas9-mediated genome editing of Epstein-Barr virus in human cells. J. Gener. Virol..

[183] Van Diemen F.R., Kruse E.M., Hooykaas M.J., Bruggeling C.E., Schurch A.C., van Ham P.M., Imhof S.M., Nijhuis M., Wiertz E.J., Lebbink R.J. (2016). CRISPR/Cas9-Mediated Genome Editing of Herpesviruses Limits Productive and Latent Infections. PLoS Pathog..

[184] Ma Y., Walsh M.J., Bernhardt K., Ashbaugh C.W., Trudeau S.J., Ashbaugh I.Y., Jiang S., Jiang C., Zhao B., Root D.E. (2017). CRISPR/Cas9 Screens Reveal Epstein-Barr Virus-Transformed B Cell Host Dependency Factors. Cell Host Microbe.

[185] Roehm P.C., Shekarabi M., Wollebo H.S., Bellizzi A., He L., Salkind J., Khalili K. (2016). Inhibition of HSV-1 Replication by Gene Editing Strategy. Sci. Rep..

[186] Bi Y., Sun L., Gao D., Ding C., Li Z., Li Y., Cun W., Li Q. (2014). High-efficiency targeted editing of large viral genomes by RNA-guided nucleases. PLoS Pathog..

[187] Moradpour D., Penin F., Rice C.M. (2007). Replication of hepatitis C virus. Nat. Rev. Microbiol..

[188] Strader D.B., Wright T., Thomas D.L., Seeff L.B. (2004). American Association for the Study of Liver, D.; Diagnosis, management, and treatment of hepatitis C. Hepatology.

[189] Andreoni M., Giacometti A., Maida I., Meraviglia P., Ripamonti D., Sarmati L. (2012). HIV-HCV co-infection: Epidemiology, pathogenesis and therapeutic implications. Eur. Rev. Med. Pharmacol. Sci..

[190] Ren Q., Li C., Yuan P., Cai C., Zhang L., Luo G.G., Wei W. (2015). A Dual-reporter system for real-time monitoring and high-throughput CRISPR/Cas9 library screening of the hepatitis C virus. Sci. Rep..

[191] Jopling C.L., Yi M., Lancaster A.M., Lemon S.M., Sarnow P. (2005). Modulation of hepatitis C virus RNA abundance by a liver-specific MicroRNA. Science.

[192] Doerflinger M., Forsyth W., Ebert G., Pellegrini M., Herold M.J. (2017). CRISPR/Cas9-The ultimate weapon to battle infectious diseases?. Cell. Microbiol..

[193] Puschnik A.S., Majzoub K., Ooi Y.S., Carette J.E. (2017). A CRISPR toolbox to study virus-host interactions. Nat. Rev. Microbiol..

[194] Zhang R., Miner J.J., Gorman M.J., Rausch K., Ramage H., White J.P., Zuiani A., Zhang P., Fernandez E., Zhang Q. (2016). A CRISPR screen defines a signal peptide processing pathway required by flaviviruses. Nature.

[195] Marceau C.D., Puschnik A.S., Majzoub K., Ooi Y.S., Brewer S.M., Fuchs G., Swaminathan K., Mata M.A., Elias J.E., Sarnow P. (2016). Genetic dissection of Flaviviridae host factors through genome-scale CRISPR screens. Nature.

[196] Savidis G., McDougall W.M., Meraner P., Perreira J.M., Portmann J.M., Trincucci G., John S.P., Aker A.M., Renzette N., Robbins D.R. (2016). Identification of Zika Virus and Dengue Virus Dependency Factors using Functional Genomics. Cell Rep..

[197] Gootenberg J.S., Abudayyeh O.O., Lee J.W., Essletzbichler P., Dy A.J., Joung J., Verdine V., Donghia N., Daringer N.M., Freije C.A. (2017). Nucleic acid detection with CRISPR-Cas13a/C2c2. Science.

[198] Ma H., Dang Y., Wu Y., Jia G., Anaya E., Zhang J., Abraham S., Choi J.G., Shi G., Qi L. (2015). A CRISPR-Based Screen Identifies Genes Essential for West-Nile-Virus-Induced Cell Death. Cell Rep..

[199] Heaton B.E., Kennedy E.M., Dumm R.E., Harding A.T., Sacco M.T., Sachs D., Heaton N.S. (2017). A CRISPR Activation Screen Identifies a Pan-avian Influenza Virus Inhibitory Host Factor. Cell Rep..

[200] Fischer W.A.N., Vetter P., Bausch D.G., Burgess T., Davey R.T., Fowler R., Hayden F.G., Jahrling P.B., Kalil A.C., Mayers D.L. (2017). Ebola virus disease: An update on post-exposure prophylaxis. Lancet Infect. Dis..

[201] Nanbo A., Watanabe S., Halfmann P., Kawaoka Y. (2013). The spatio-temporal distribution dynamics of Ebola virus proteins and RNA in infected cells. Sci. Rep..

[202] Kennedy S.B., Bolay F., Kieh M., Grandits G., Badio M., Ballou R., Eckes R., Feinberg M., Follmann D., Grund B. (2017). Phase 2 Placebo-Controlled Trial of Two Vaccines to Prevent Ebola in Liberia. N. Engl. J. Med..

[203] Cote M., Misasi J., Ren T., Bruchez A., Lee K., Filone C.M., Hensley L., Li Q., Ory D., Chandran K. (2011). Small molecule inhibitors reveal Niemann-Pick C1 is essential for Ebola virus infection. Nature.

[204] Miller E.H., Obernosterer G., Raaben M., Herbert A.S., Deffieu M.S., Krishnan A., Ndungo E., Sandesara R.G., Carette J.E., Kuehne A.I. (2012). Ebola virus entry requires the host-programmed recognition of an intracellular receptor. EMBO J..

[205] Carette J.E., Raaben M., Wong A.C., Herbert A.S., Obernosterer G., Mulherkar N., Kuehne A.I., Kranzusch P.J., Griffin A.M., Ruthel G. (2011). Ebola virus entry requires the cholesterol transporter Niemann-Pick C1. Nature.

[206] Kondratowicz A.S., Lennemann N.J., Sinn P.L., Davey R.A., Hunt C.L., Moller-Tank S., Meyerholz D.K., Rennert P., Mullins R.F., Brindley M. (2011). T-cell immunoglobulin and mucin domain 1 (TIM-1) is a receptor for Zaire Ebolavirus and Lake Victoria Marburgvirus. Proc. Natl. Acad. Sci. USA.

[207] Younan P., Iampietro M., Nishida A., Ramanathan P., Santos R.I., Dutta M., Lubaki N.M., Koup R.A., Katze M.G., Bukreyev A. (2017). Ebola Virus Binding to Tim-1 on T Lymphocytes Induces a Cytokine Storm. mBio.

[208] Chen Y., Cai H., Pan J., Xiang N., Tien P., Ahola T., Guo D. (2009). Functional screen reveals SARS coronavirus nonstructural protein nsp14 as a novel cap N7 methyltransferase. Proc. Natl. Acad. Sci. USA.

[209] Rota P.A., Oberste M.S., Monroe S.S., Nix W.A., Campagnoli R., Icenogle J.P., Penaranda S., Bankamp B., Maher K., Chen M.H. (2003). Characterization of a novel coronavirus associated with severe acute respiratory syndrome. Science.

[210] Li W., Moore M.J., Vasilieva N., Sui J., Wong S.K., Berne M.A., Somasundaran M., Sullivan J.L., Luzuriaga K., Greenough T.C. (2003). Angiotensin-converting enzyme 2 is a functional receptor for the SARS coronavirus. Nature.

[211] Kuba K., Imai Y., Rao S., Gao H., Guo F., Guan B., Huan Y., Yang P., Zhang Y., Deng W. (2005). A crucial role of angiotensin converting enzyme 2 (ACE2) in SARS coronavirus-induced lung injury. Nat. Med..

[212] Imai Y., Kuba K., Rao S., Huan Y., Guo F., Guan B., Yang P., Sarao R., Wada T., Leong-Poi H. (2005). Angiotensin-converting enzyme 2 protects from severe acute lung failure. Nature.

[213] Huang I.C., Bailey C.C., Weyer J.L., Radoshitzky S.R., Becker M.M., Chiang J.J., Brass A.L., Ahmed A.A., Chi X., Dong L. (2011). Distinct patterns of IFITM-mediated restriction of filoviruses, SARS coronavirus, and influenza A virus. PLoS Pathog..

[214] Raj V.S., Mou H., Smits S.L., Dekkers D.H., Muller M.A., Dijkman R., Muth D., Demmers J.A., Zaki A., Fouchier R.A. (2013). Dipeptidyl peptidase 4 is a functional receptor for the emerging human coronavirus-EMC. Nature.

[215] Lu G., Hu Y., Wang Q., Qi J., Gao F., Li Y., Zhang Y., Zhang W., Yuan Y., Bao J. (2013). Molecular basis of binding between novel human coronavirus MERS-CoV and its receptor CD26. Nature.

[216] Wang N., Shi X., Jiang L., Zhang S., Wang D., Tong P., Guo D., Fu L., Cui Y., Liu X. (2013). Structure of MERS-CoV spike receptor-binding domain complexed with human receptor DPP4. Cell Res..

[217] Earnest J.T., Hantak M.P., Li K., McCray P.B., Perlman S., Gallagher T. (2017). The tetraspanin CD9 facilitates MERS-coronavirus entry by scaffolding host cell receptors and proteases. PLoS Pathog..

[218] Mansoor S., Zafar Y., Briddon R.W. (2006). Geminivirus disease complexes: The threat is spreading. Trends Plant Sci..

[219] Hanley-Bowdoin L., Bejarano E.R., Robertson D., Mansoor S. (2013). Geminiviruses: Masters at redirecting and reprogramming plant processes. Nat. Rev. Microbiol..

[220] Yang C.F., Chen K.C., Cheng Y.H., Raja J.A., Huang Y.L., Chien W.C., Yeh S.D. (2014). Generation of marker-free transgenic plants concurrently resistant to a DNA geminivirus and a RNA tospovirus. Sci. Rep..

[221] Varsani A., Navas-Castillo J., Moriones E., Hernandez-Zepeda C., Idris A., Brown J.K., Murilo Zerbini F., Martin D.P. (2014). Establishment of three new genera in the family Geminiviridae: Becurtovirus, Eragrovirus and Turncurtovirus. Arch. Virol..

[222] Ji X., Zhang H., Zhang Y., Wang Y., Gao C. (2015). Establishing a CRISPR-Cas-like immune system conferring DNA virus resistance in plants. Nat. Plants.

[223] Ali Z., Abulfaraj A., Idris A., Ali S., Tashkandi M., Mahfouz M.M. (2015). CRISPR/Cas9-mediated viral interference in plants. Genome Biol..

[224] Ali Z., Ali S., Tashkandi M., Zaidi S.S., Mahfouz M.M. (2016). CRISPR/Cas9-Mediated Immunity to Geminiviruses: Differential Interference and Evasion. Sci. Rep..

[225] Baltes N.J., Hummel A.W., Konecna E., Cegan R., Bruns A.N., Bisaro D.M., Voytas D.F. (2015). Conferring resistance to geminiviruses with the CRISPR—Cas prokaryotic immune system. Nat. Plants.

[226] Hutter G., Bodor J., Ledger S., Boyd M., Millington M., Tsie M., Symonds G. (2015). CCR5 Targeted Cell Therapy for HIV and Prevention of Viral Escape. Viruses.

[227] Afkhami S., Yao Y., Xing Z. (2016). Methods and clinical development of adenovirus-vectored vaccines against mucosal pathogens. Mol. Ther. Methods Clin. Dev..

[228] Wold W.S., Toth K. (2013). Adenovirus vectors for gene therapy, vaccination and cancer gene therapy. Curr. Gene Ther..

[229] Wang C.X., Cannon P.M. (2016). The clinical applications of genome editing in HIV. Blood.

[230] Weatherley D.A.V., Boswell M.T., Rowland-Jones S.L. (2017). Targeting TRIM5alpha in HIV Cure Strategies for the CRISPR-Cas9 Era. Front. Immunol..

[231] Tebas P., Stein D., Tang W.W., Frank I., Wang S.Q., Lee G., Spratt S.K., Surosky R.T., Giedlin M.A., Nichol G. (2014). Gene editing of CCR5 in autologous CD4 T cells of persons infected with HIV. N. Engl. J. Med..

